# RNA-seq analysis of hepatic gene expression of common Pekin, Muscovy, mule and hinny ducks fed ad libitum or overfed

**DOI:** 10.1186/s12864-018-5415-1

**Published:** 2019-01-07

**Authors:** Frédéric Hérault, Magalie Houée-Bigot, Elisabeth Baéza, Olivier Bouchez, Diane Esquerré, Christophe Klopp, Christian Diot

**Affiliations:** 1 PEGASE, INRA, Agrocampus Ouest, 16 Le Clos, 35590 Saint-Gilles, France; 2 Mathématiques Appliquées, Agrocampus Ouest, 35042 Rennes, France; 3 BOA, INRA, Université de Tours, 37380 Nouzilly, France; 4 GENPhySE, INRA, ENVT, ENSAT, 31326 Castanet-Tolosan, France; 5 GeT PlaGE, INRA, US 1426, GenoToul, 31326 Castanet-Tolosan, France; 6 Bioinformatics facility, INRA, Genotoul, 31326 Castanet-Tolosan, France; 7 SIGENAE, INRA, 31326 Castanet-Tolosan, France

**Keywords:** RNA sequencing, Differentially expressed genes, Overfeeding, Hepatic steatosis, Ducks

## Abstract

**Background:**

Duck species are known to have different susceptibility to fatty liver production in response to overfeeding. In order to better describe mechanisms involved in the development of hepatic steatosis and differences between species, transcriptome analyses were conducted on RNAs extracted from the livers of Pekin and Muscovy duck species and of their reciprocal hybrids, Mule and Hinny ducks fed ad libitum or overfed to identify differentially expressed genes and associated functions.

**Results:**

After extraction from the liver of ducks from the four genetic types, RNAs were sequenced and sequencing data were analyzed. Hierarchic clustering and principal component analyses of genes expression levels indicated that differences between individuals lie primarily in feeding effect, differences between genetic types being less important. However, Muscovy ducks fed ad libitum and overfed were clustered together. Interestingly, Hinny and Mule hybrid ducks could not be differentiated from each other, according to feeding. Many genes with expression differences between overfed and ad libitum fed ducks were identified in each genetic type. Functional annotation analyses of these differentially expressed genes highlighted some expected functions (carbohydrate and lipid metabolisms) but also some unexpected ones (cell proliferation and immunity).

**Conclusions:**

These analyses evidence differences in response to overfeeding between different genetic types and help to better characterize functions involved in hepatic steatosis in ducks.

**Electronic supplementary material:**

The online version of this article (10.1186/s12864-018-5415-1) contains supplementary material, which is available to authorized users.

## Background

In western human populations non-alcoholic fatty liver disease (NAFLD), i.e. fat accumulation in the liver, represents the most common cause of abnormal liver function [1]. This hepatic steatosis is associated with overeating of energy-rich food (e.g. high sugar or high fat diet). In terms of human health, NAFLD is frequently associated with various forms of metabolic disorders including obesity, insulin resistance and type 2 diabetes. Increased fatty acids (FA) hepatic synthesis or de novo lipogenesis is known as one major cause of hepatic steatosis and is linked to overexpression of lipogenic genes [2–5]. In addition, hepatic steatosis is also linked to increased FA uptake, decreased FA β-oxidation and/or decreased synthesis or secretion of very low density lipoproteins (VLDLs) [6, 7]. Expression of adipokines was also demonstrated to play a role in the development of hepatic steatosis [8–10]. From a clinical point of view, NAFLD is the early step of a sequence of syndromes including steatohepatitis (NASH), fibrosis and cirrhosis and is now considered an inducer of many distinct injurious factors [11]. It is also known that patients with hepatic steatosis do not always develop necroinflammation or liver fibrosis [12] indicating individual variability in liver disease susceptibility due to genetic and/or environmental factors [13, 14]. Altogether, these results indicate that hepatic steatosis is the result of a large number of metabolic processes, gene expressions and factors that still need to be characterized.

Recently, it was suggested that NAFLD and hepatic steatosis in overfed birds are very similar [15]. Indeed, liver steatosis can occur spontaneously in wild waterfowls as a result of energy storage before migration. This ability is exploited since thousand years in domesticated birds to produce “foie gras” by overfeeding. In these birds, hepatic steatosis exists in isolation, a degenerative event rarely occurs, and it is reversible when overfeeding is stopped [16]. Genetic effects are also known to play a role in waterfowl ability to produce fatty liver [16–20]. Some evidence suggests that the genetic differences observed in liver steatosis ability between different duck breeds or species are in some part the result of differences in de novo lipogenesis [20], lipoprotein assembly and secretion [16, 20] and extrahepatic uptake of plasma lipids [19]. Until recently, most of these studies focused on the expression of some genes involved in lipid metabolism as tools for duck genome-wide gene expression were unavailable. It was thus demonstrated that genes involved in carbohydrates and lipid metabolisms were regulated by overfeeding in ducks [21, 22].

The recent emergence of next generation sequencing (NGS) techniques allows the analysis of genes expressed from a whole genome by RNA sequencing. The present study was thus conducted to analyze genome-wide gene expression in Muscovy ducks (*Cairina moschata*) and common Pekin (*Anas platyrhynchos*) duck species and in their reciprocal inter-specific hybrids, i.e. Mule ducks (the sterile hybrid from a male Muscovy duck and a female common duck) and Hinny ducks (the sterile hybrid from a male common duck and a female Muscovy duck). Analysis of differentially expressed genes in these 4 genetic types and comparison between species were also conducted in order to evidence similarities and differences of responses to overfeeding in these genetic types that are (Muscovy and Mule ducks) or not (Pekin and Hinny ducks) used for “foie gras” production.

## Methods

### Animals and experimental design

Animals and experimental design have been described previously [20]. Briefly and as described in this publication, male ducks corresponding to common Pekin (*Anas platyrhynchos*) and Muscovy (*Cairina moschata*) duck species and to their two reciprocal interspecific Mule and Hinny hybrids (24 per genotype) were provided by Grimaud (Roussay, France). They were reared under usual conditions of light and temperature at the Experimental Station for Waterfowl Breeding (INRA Artiguères, France). From hatching to 6 weeks of age, they were fed ad libitum. From 6 to 12 weeks of age, they were fed on a restricted diet at levels appropriate for each genotype (200–250 g per duck at the beginning, increasing to 360–380 g at the end of the period). At 12 weeks of age, ducks were either fed ad libitum with the growing diet (controls) or overfed 14 days with high carbohydrate corn and corn meal. This overfeeding with high carbohydrate diet is referred thereafter as overfeeding. Ducks were slaughtered 14 h after the last meal by electronarcosis, neck sectioning and bleeding. Immediately after bleeding, liver were weighted and sampled, rapidly frozen in liquid nitrogen and stored at − 80 °C. These liver samples were kindly provided by Baéza and Chartrin [20] and reused in the present study for RNA sequencing and expression analyses.

### RNA preparation and sequencing

Total RNA was extracted from 96 liver samples (12 ducks per genetic type and per diet) using NucleoSpin® RNA L (Macherey-Nagel SARL, Hoerdt, France) according to the manufacturer’s instructions. This kit involves guanidinium thiocyanate, silica membrane and on-column RNase-free DNase digestion. RNA concentration was determined using a ND-1000 Spectrophotometer (Thermo Scientific, Illkirch, France). Integrity of RNA was checked with Lab-on-a-Chip Eukaryote Total RNA Nano and Bionalyzer 2100 (Agilent Technologies France, Massy, France). 79 RNA with absorbance ratio λ260nm/λ280nm and λ260nm/λ230nm > 1.8 and RNA integrity number [23] or RIN > 7.4 were selected (9–10 ducks per genetic type and per diet).

Libraries preparation and sequencing were performed at GeT Plage, the genomics facility of Genotoul (http://get.genotoul.fr/en/). RNA-seq paired-end libraries have been prepared according to Illumina’s protocols using the Illumina TruSeq RNA Sample Prep Kit v2 to analyze mRNA (Illumina, San Diego, CA). Briefly, mRNA were selected using poly-T beads. Then, RNA were fragmented to generate double stranded cDNA and adaptators were ligated to be sequenced. 10 cycles of PCR were applied to amplify libraries. Library quality was assessed using an Agilent Bioanalyser (Agilent Technologies France, Massy, France) and libraries were quantified by qPCR using the Kapa Library Quantification Kit. RNA-seq experiments were performed on an Illumina HiSeq2000 using a paired-end read length of 2 × 100 pb with the Illumina HiSeq2000 SBS v3 sequencing kits. Samples were tagged using hexamer Tag sequences for subsequent identification (Additional file 1). The libraries were sequenced in paired-ends on 14 different lanes, 6 samples per lane (Additional file 1).

Sequence data were submitted to the NCBI sequence read archive (SRA) under the accession number SRP144764.

### Sequence analyses

Raw sequences pre-treatments and analyses were performed on Bioinformatics facility of Genotoul (http://bioinfo.genotoul.fr/). After FastQC quality check (http://www.bioinformatics.babraham .ac.uk/projects/fastqc/), adapter trimming, and merging sample sequences, RNA sequences were aligned with STAR aligner (version 2.3.0e [24] on the common duck (*Anas platyrhynchos*) reference genome (BGI_duck_1.0, INSDC Assembly GCA_000355885.1). A similar approach was applied in a study where horse, donkey and reciprocal hybrids RNA reads were mapped against the horse reference genome [25]. Cufflinks and Cuffmerge tools [26] were then applied on the merged file to construct a new gene model file. Gene raw counts (Additional files 2 and 3) were obtained using featureCounts (version 1.4.5-p1 using -s 2 -O -p -t exon -g gene_id parameters).

### Gene expression analyses

Gene expression analyses were performed using R software version 3.2.2 (https://www.R-project.org). Gene expressions were determined using high-quality reads. Expression data (Additional files 2 and 3) were first normalized with the Trimmed Mean of M-values (TMM) method [27] using the Bioconductor package edgeR [28]. Normalized data were analyzed by Principal component analyses and hierarchical clustering. Genes were filtered using the data driven HTSfilter procedure using the Bioconductor package HTSFilter [29]. Differentially expressed genes (DEG) between overfed and ad libitum fed ducks were determined in each genetic type using filtered genes set.

### Functional annotation

Functional annotation of differentially expressed genes with assigned GO terms for biological processes (GOBP) was conducted with the Database for Annotation, Visualization and Integrated Discovery (DAVID, http://david.abcc.ncifcrf.gov/) bioinformatics resource, version 6.82016 [30–32]. Enrichment of functional annotations in DEG was determined using the default medium classification stringency with an EASE enrichment threshold (a modified Fisher Exact *P*-Value) set to 0.001. Enriched annotations were then clustered with the Wang method [33] using GO terms semantic similarity as metric. Expression profiles of down- and up-regulated DEG in the different clusters were also analyzed and visualized using clusterProfiler [34]. Dot size in profiles corresponds to FC. *P*-values are shown in color bar, values decrease from more (red) to less significant (blue).

### Gene interaction networks

Gene interaction networks were determined using the STRING database of known and predicted protein-protein interactions (https://string-db.org/) version 10.5 [35, 36]. Minimum required interaction score was set to medium confidence (0.400).

## Results

### Phenotypic data

As previously described [20], overfeeding induced a significant increase in liver weight, especially in Mule, Hinny and Muscovy ducks and less in Pekin ducks when compared to fed ad libitum control ducks (Additional file 4). This liver weight increase was correlated to increased lipid and triglyceride levels and decreased water and protein levels in the liver. These data indicated that hepatic steatosis occurred more or less in overfed ducks from the 4 genetic types.

### RNA sequencing and expression data

**Fig. 1:**
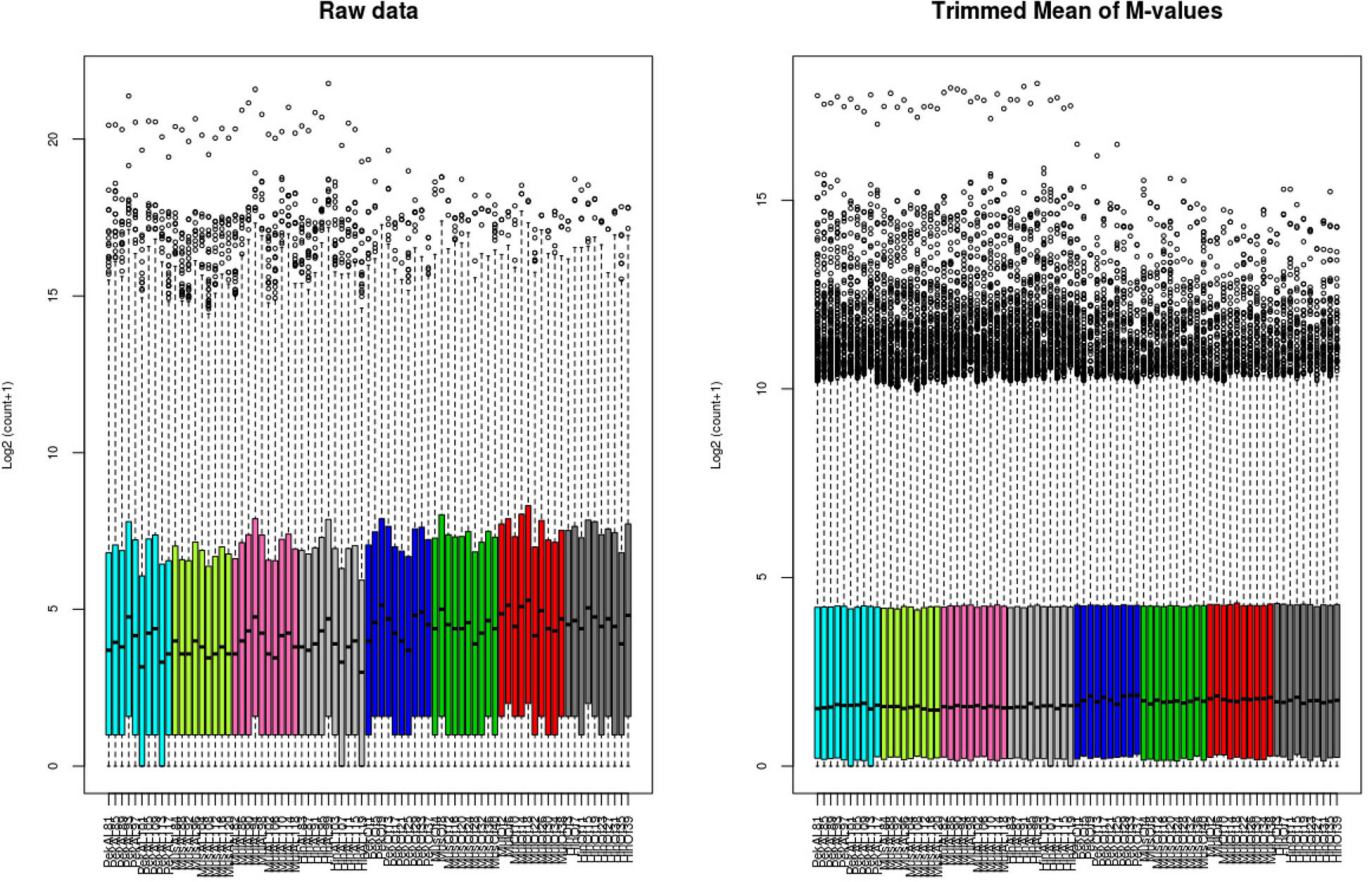
Normalization of raw data. Boxplot distribution of read counts (log2 (counts+ 1)) in each sample before (left panel) and after normalization by TMM method (right panel). Light blue: Pekin ducks fed ad libitum; dark blue: overfed Pekin ducks; light green: Muscovy ducks fed ad libitum; dark green: overfed Muscovy ducks; pink: Mule ducks fed ad libitum; red: overfed Mule ducks; light grey: Hinny ducks fed ad libitum; dark grey: overfed Hinny ducks

To go further in the understanding of mechanisms involved in hepatic steatosis, gene expression analyses were conducted by high-throughput RNA sequencing. For this purpose, RNA were first extracted from the liver of Pekin, Muscovy, Mule and Hinny ducks fed ad libitum or overfed and were sequenced on a HiSeq2000 after cDNA library constructions (*n* = 79). Approximately 2215 million paired-ends sequences were produced, corresponding to 28 ± 8 million in each sample and to 27.6 ± 7.7 million (97.4 to 99.1%) high-quality paired-reads (Additional file 1). High-quality reads were assembled in 22,561 expressed genes and for each of these genes the number of mapped reads in each sample was counted (Additional files 2 and 3). These counts were considered raw expression data. To exclude biases linked to different sample preparations, raw data were normalized using the TMM method (Fig. 1).

### Comparison of duck liver transcriptomes

**Fig. 2:**
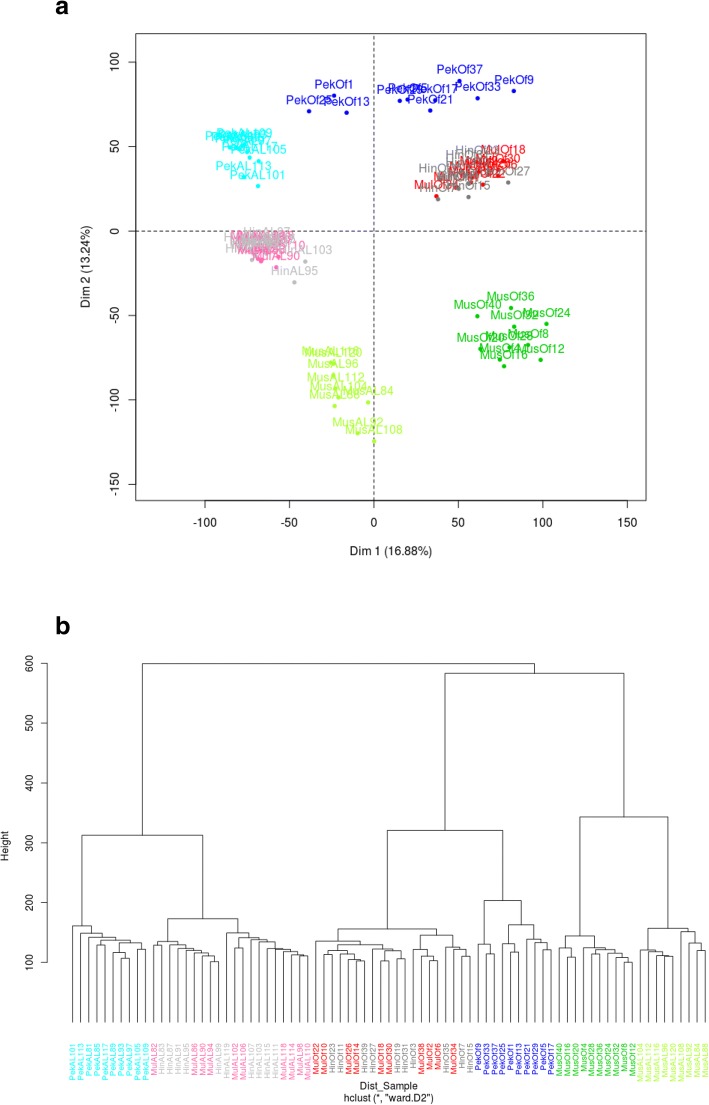
Clustering of duck samples according to gene expression. Comparison of gene expression in duck samples by principal component analysis (**a**) and hierarchical clustering (**b**). Legend of the samples is indicated in Fig. 1

Normalized expression data were analyzed by principal component analysis (PCA) and hierarchical clustering (HC) in order to compare the liver transcriptomes. Six clusters were clearly defined by PCA (Fig. 2a). The first principal component (Dim 1) summarized 17% of the whole variability and discriminated samples according to feeding (overfed versus ad libitum). The second principal component (Dim 2) summarized 13% of the whole variability and discriminated samples according to genetic type, pure species being extreme and hybrids intermediate. It could be observed that the cluster corresponding to overfed Pekin ducks appeared more dispersed than other clusters and that Mule and Hinny samples were clustered together according to feeding (ad libitum and overfed). Hierarchical clustering of normalized data (Fig. 2b) produced very similar results, samples were first clustered according to feeding and hybrid ducks with the same feeding were clustered together. However, it appeared that Muscovy ducks fed ad libitum or overfed were clustered together.

### Differential gene expression analyses

**Fig. 3:**
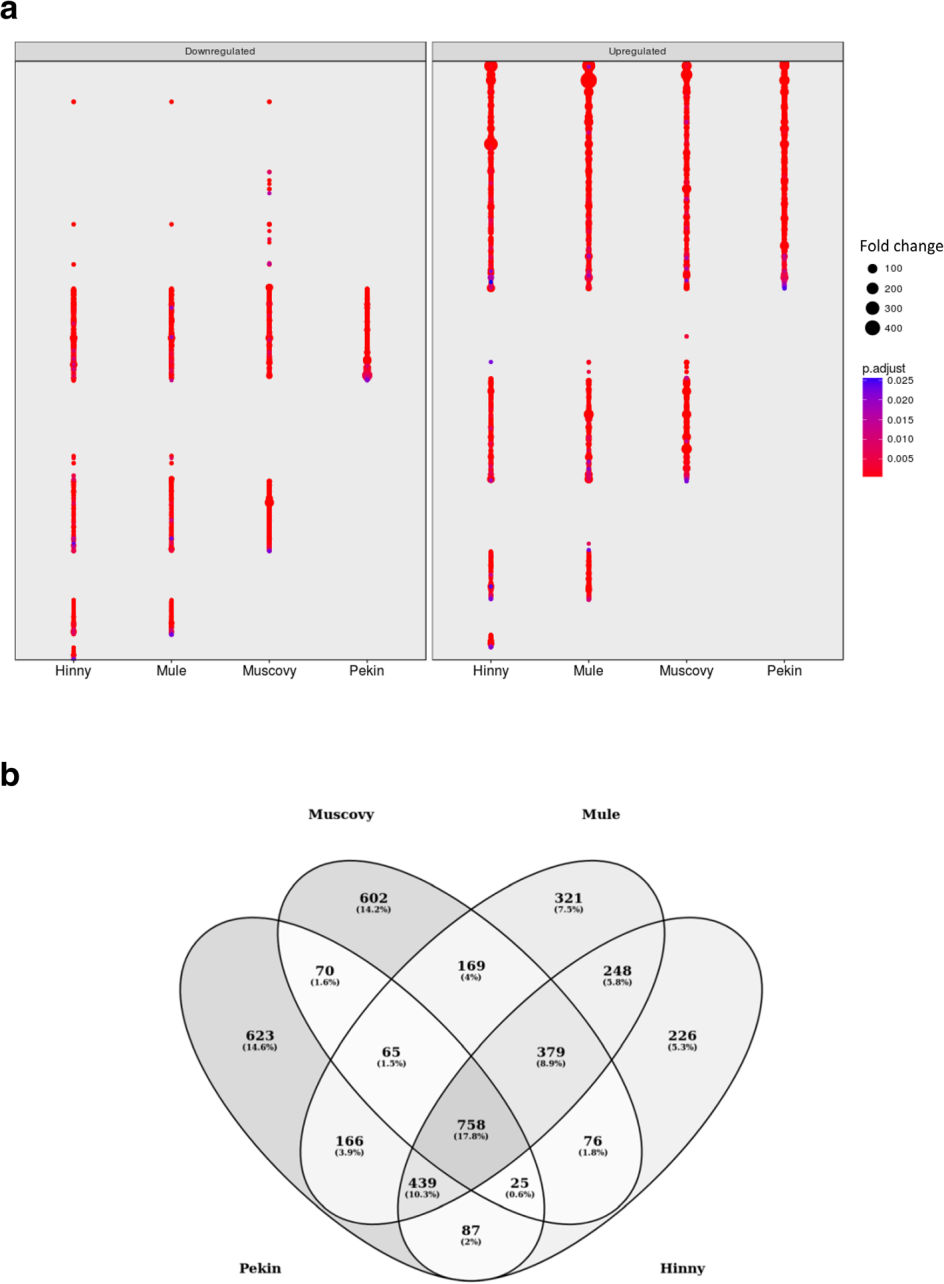
Differentially expressed genes. Fold changes (FC > 2) of down- (left panel) and up-regulated (right panel) significant (adjusted *p* value < 0.05) differentially expressed genes (DEG) in the four genetic types (**a**). Venn diagram of DEG in the four genetic types (**b**)

**Fig. 4:**
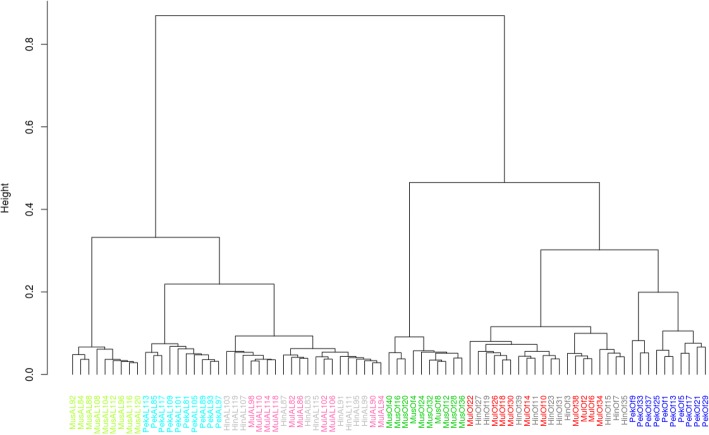
Hierarchical clustering of duck samples according to differential gene expression. Legend of the samples is indicated in Fig. 1

In order to better describe the different samples, differences in gene expressions between fed ad libitum and overfed ducks were analyzed. For each genetic type, genes showing a significant difference (*p* < 0.05) and a fold change ≥2 were selected (Fig. 3a). As indicated in Table 1, 2233, 2144, 2545 and 2238 genes were found up-and down-regulated by overfeeding, i.e. differentially expressed genes (DEG), in Pekin, Muscovy, Mule and Hinny ducks, respectively. Some of these genes were previously identified by RT-PCR in the same Pekin and Muscovy duck liver samples [21, 37]. Although only 758 DEG (17.8%) were common to the 4 genetic types, 1824 were found between Mule and Hinny hybrids, 918 between the Pekin and Muscovy ducks, and approximately the mean of these 2 DEG numbers between one hybrid and one duck species, 1238, 1309, 1374 and 1428 between Hinny and Muscovy, Hinny and Pekin, Mule and Pekin and Mule and Muscovy, respectively (Fig. 3b). Hierarchical clustering (HC) was then conducted using all DEG expression data (Fig. 4). Again, samples were first clustered according to feeding, and then according to genetic type.

**Table 1:** Differentially expressed genes

DEG	Pekin	Muscovy	Mule	Hinny	common
up-regulated	1553	1371	1592	1314	520
down-regulated	680	773	953	924	235
all	2233	2144	2545	2238	758

### Functional annotation of differentially expressed genes

**Fig. 5:**
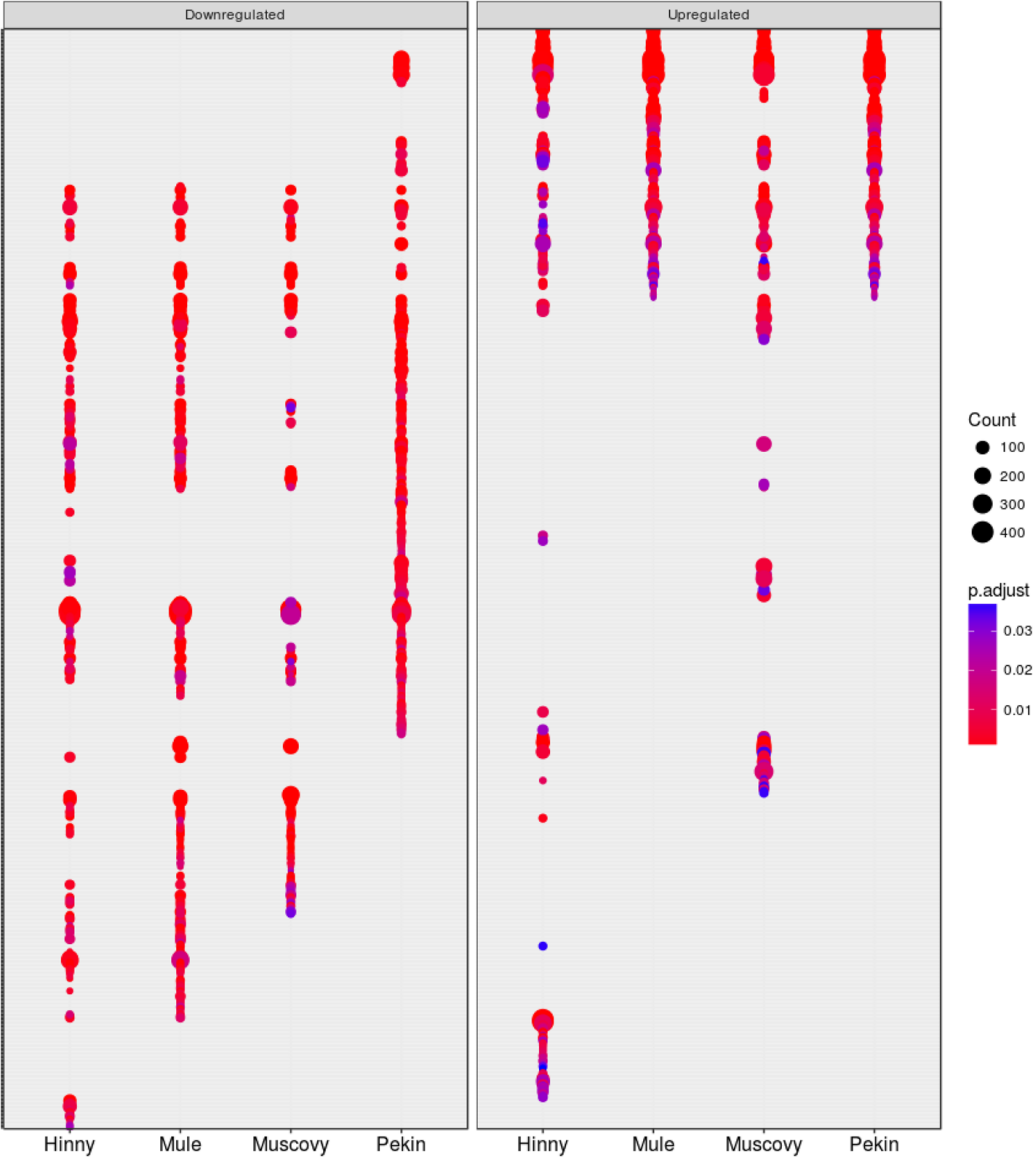
Enriched annotation profiles associated to differentially expressed genes. Dot representation of 611 significant (*p* < 0.05) enriched GO terms associated to down- (left panel) and up-regulated (right panel) differentially expressed genes (DEG). Count indicates the number of DEG annotated with the GO term

**Fig. 6:**
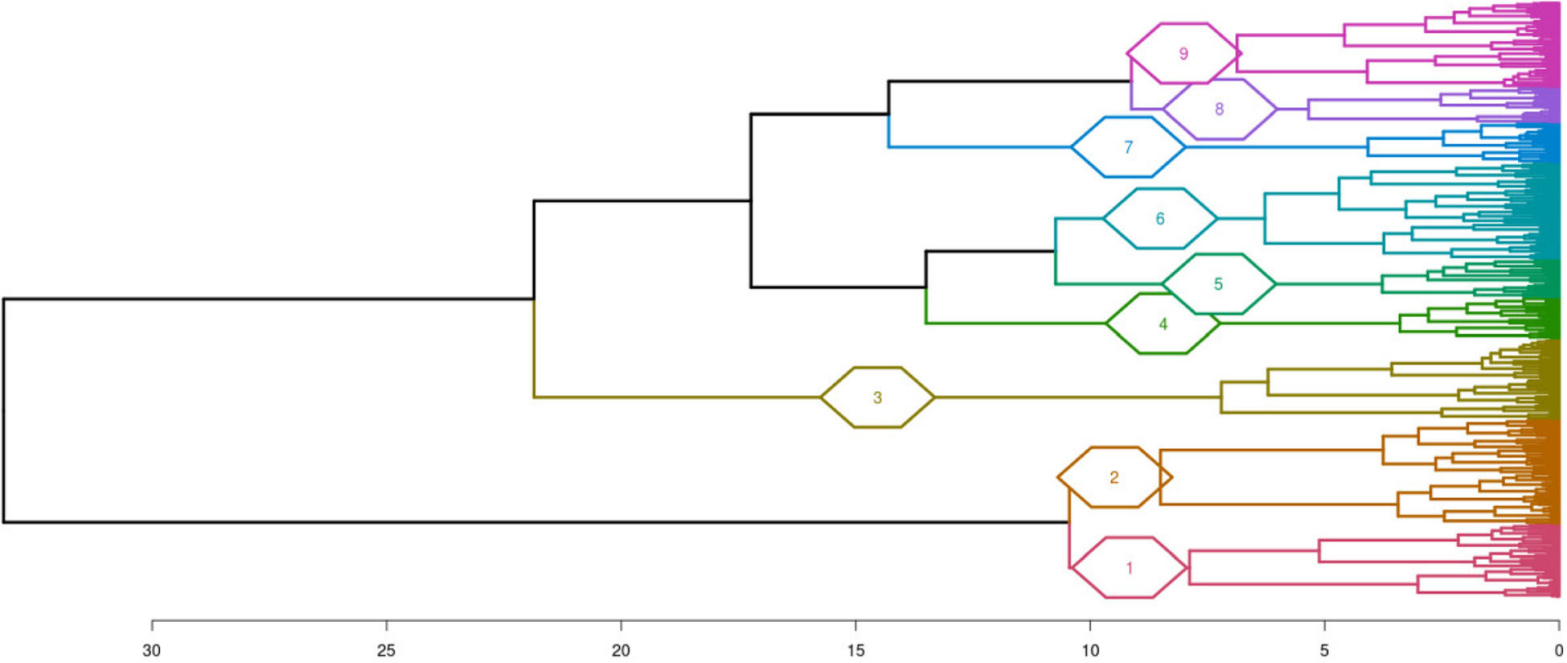
Semantic similarity clustering of enriched GO terms associated to differentially expressed genes. The 611 enriched GO terms were clustered according to their semantic similarity using the method of Wang. Cluster 1: “Cellular aromatic compound metabolic process” including 75 GO terms; Cluster 2: “Organic acid metabolic process” (108 GO terms); Cluster 3: “Anatomical structure development” (83 GO terms); Cluster 4: “Response to organic substance” (42 GO terms); Cluster 5: “Organic substance metabolic process” (40 GO terms); Cluster 6: “Regulation of biological process” (99 GO terms); Cluster 7: “Transport” (41 GO terms); Cluster 8: “Cellular component organization” (36 GO terms); Cluster 9: “Cell cycle process” (87 GO terms). GO terms in each cluster are indicated in Additional file 5

Enriched functional annotations in DEG were determined with DAVID and clusterProfiler annotation tools. A large number of biological processes (611 GO terms) associated to DEG were found enriched, either up-or down-regulated by overfeeding, drawing enriched annotation profiles (EAP) (Fig. 5). These EAP allow showing down- and up-regulated responses to overfeeding in the 4 genetic types in an easy way for comparison. Some similarities and particularities between species are visualized, for example similarities in Mule and Pekin up-regulated functions or Muscovy, Mule and Hinny down-regulated functions. To describe these annotations in a more synthetic way, the 611 enriched terms were clustered according to semantic similarity (Fig. 6). Nine clusters were defined, grouping 183 terms in 2 metabolic process clusters (clusters 1 and 2) and 428 terms in 7 cellular process clusters (clusters 3–9). For each of these clusters an EAP was drawn (Additional file 5).

**Fig. 7:**
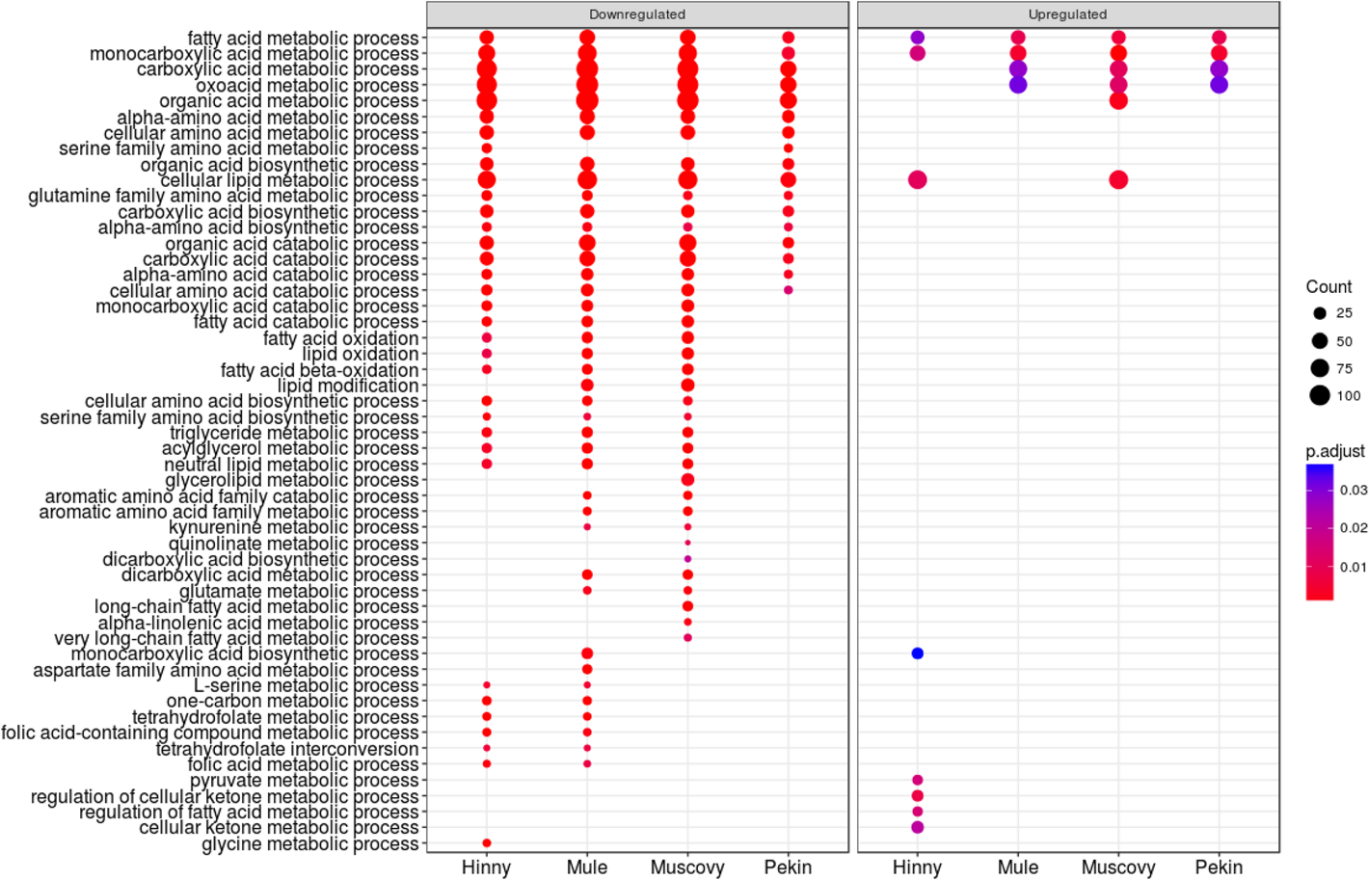
Enriched GO terms associated to differentially expressed genes in “Organic acid metabolic process” cluster 2. Dot representation of significant (p < 0.05) enriched GO terms associated to down- (left panel) and up-regulated (right panel) differentially expressed genes (DEG)and corresponding to the lower part of cluster 2 (see Additional file 5 for a complete view of cluster 2). Count indicates the number of DEG annotated with the same GO term

**Fig. 8:**
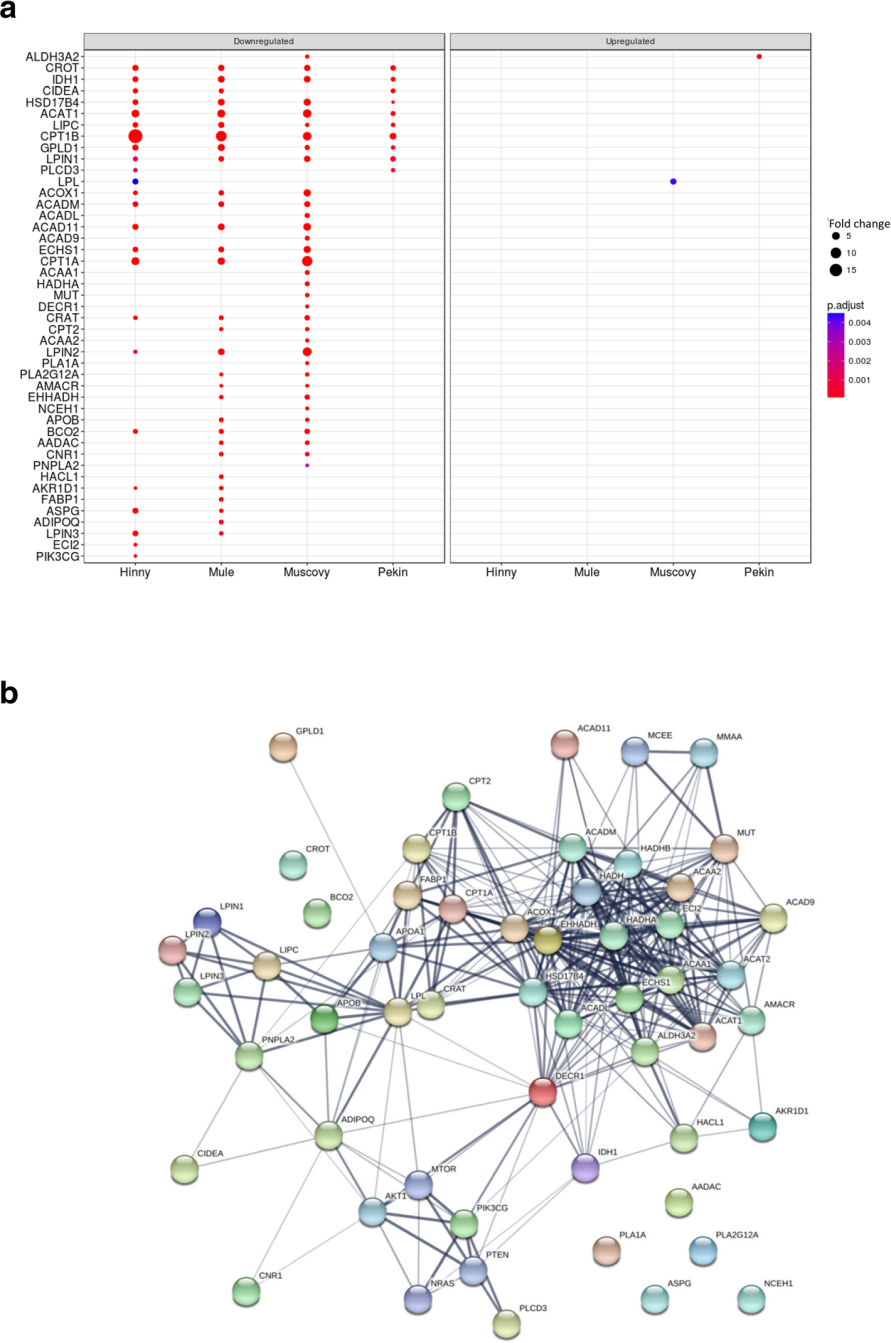
DEG involved in “lipid catabolic process”. Fold changes (FC > 2) in the four genetic types of the 45 down- (left panel) and up-regulated (right panel) significant (adjusted p value < 0.05) differentially expressed genes (DEG) annotated with lipid catabolism GO term (**a**). Interaction network of these 45 genes as defined by STRING (**b**). Minimum required interaction score was set to medium confidence (0.400). Lline thickness of the edges indicates the strength of data support (fusion, neighborhood, co-occurrence, experimental, text-mining, database and co-expression evidences)

As expected, lipid metabolic process was enriched (Additional file 5, cluster 2). Interestingly, lipid oxidation, fatty acid oxidation and fatty acid beta−oxidation were also enriched, down-regulated in the liver of Hinny, Mule and Muscovy overfed ducks (Fig. 7). Fatty acid beta−oxidation enrichment resulted from down-regulation of 45 genes (Fig. 8a). Interaction network of these genes was analyzed (Fig. 8b). The network had significantly more interactions than expected (298 edges in the network versus 55 expected) again suggesting that these genes jointly contribute to a shared function.

**Fig. 9:**
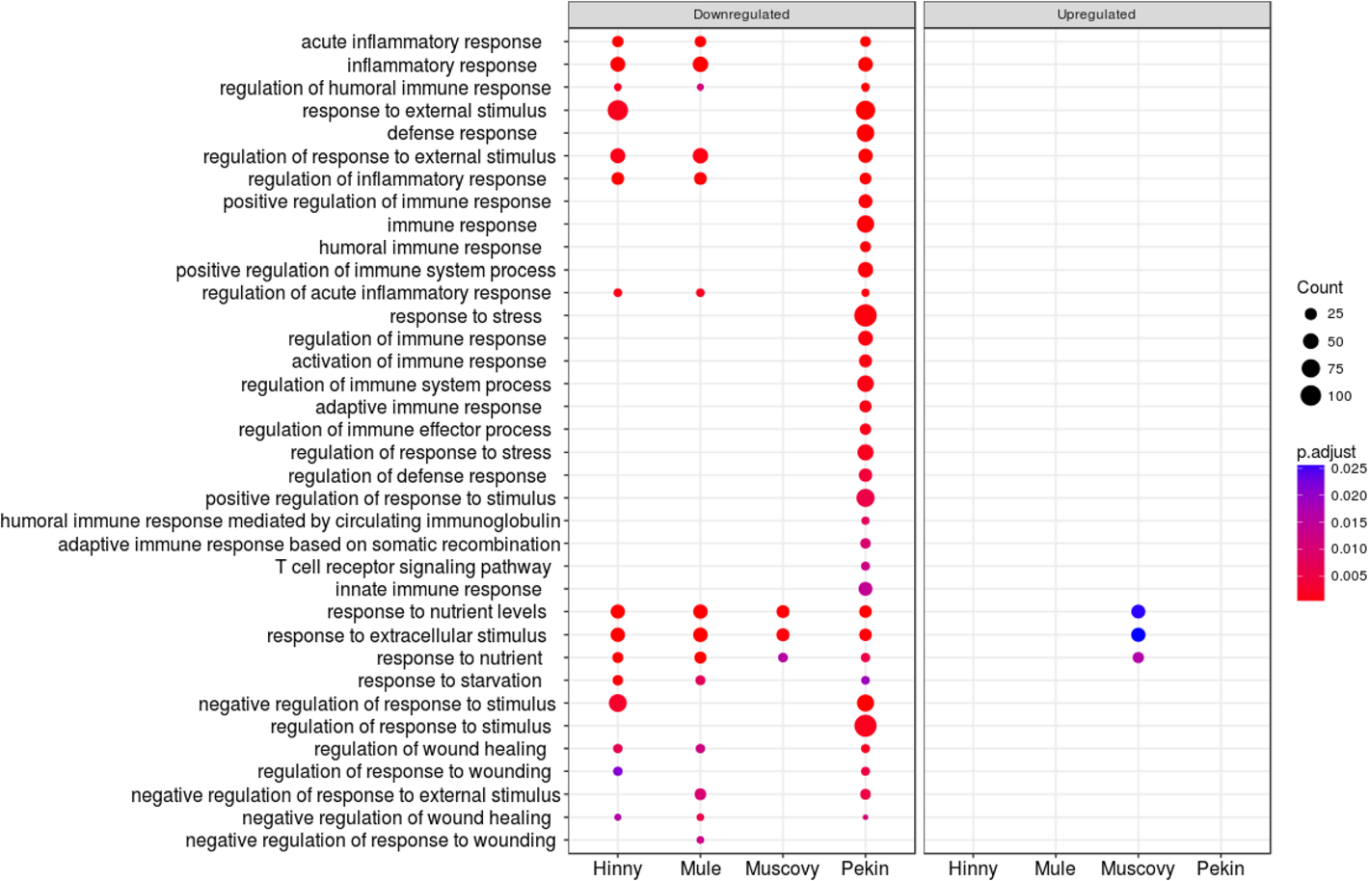
Enriched GO terms associated to differentially expressed genes in “Regulation of biological process” cluster 6. Dot representation of significant (p < 0.05) enriched GO terms associated to down- (left panel) and up-regulated (right panel) differentially expressed genes (DEG)and corresponding to the lower part of cluster 6 (cluster 6.1 in Additional file 5). Count indicates the number of DEG annotated with the same GO term

**Fig. 10:**
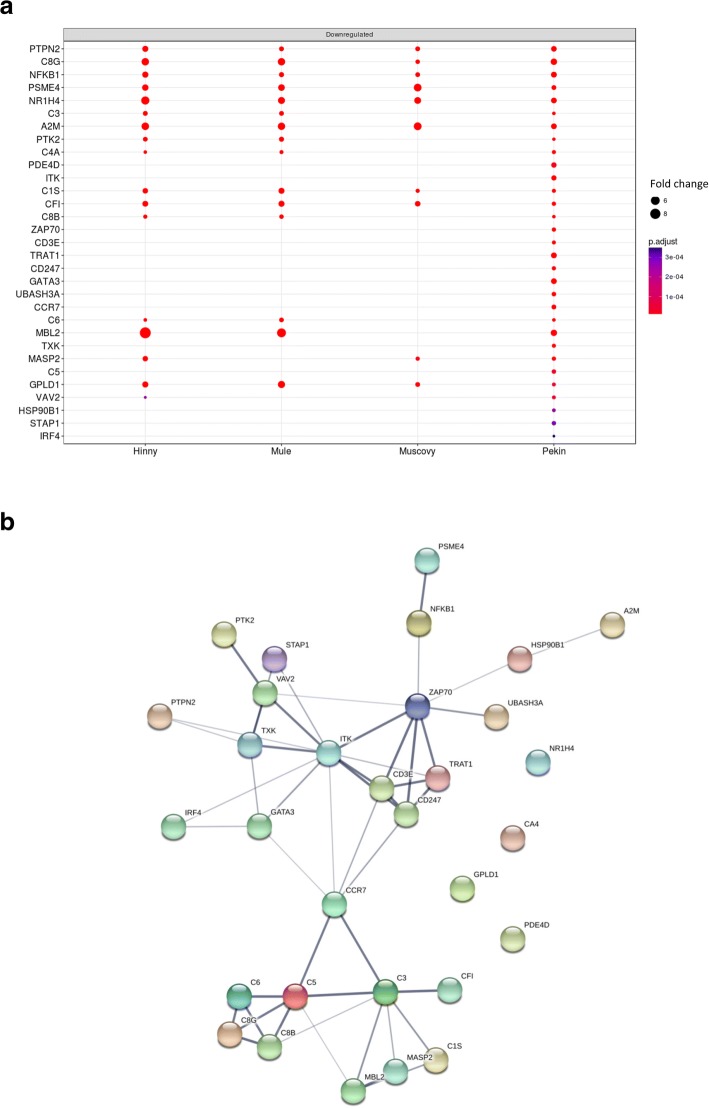
DEG involved in “activation of immune response”. Fold changes (FC > 2) in the four genetic types of the 31 down-regulated significant (adjusted p value < 0.05) differentially expressed genes (DEG) annotated with “activation of immune response” GO term (**a**). Interaction network of the 31 genes as defined by STRING (b). Minimum required interaction score was set to medium confidence (0.400). Line thickness of the edges indicates the strength of data support (fusion, neighborhood, co-occurrence, experimental, text-mining, database and co-expression evidences) (**b**)

Some unexpected or yet not described processes were also enriched in DEG, for example those associated to cell cycle, extra-cellular matrix organization, blood coagulation and immune response (Additional file 5). Enriched regulation of biological process (Additional file 5, cluster 6.1) included many down-regulated terms associated to immune response (Fig. 9). Among them, enrichment of activation of immune response term resulted from down-regulation of 31 genes especially in Pekin ducks (Fig. 10a) with more interactions (49) than expected (8) (Fig. 10b).

## Discussion

Some studies have been conducted in duck species and more generally in waterfowls in order to describe hepatic steatosis development after overfeeding. Due to the lack of specific microarrays, most of them were conducted by analyzing the expression of few candidate genes, especially those known to play a role in lipid and carbohydrate metabolisms [21, 22, 38]. The recent development of next generation sequencing (NGS) techniques allows analysis of all the genes expressed from a genome. In this context, we have conducted RNA sequencing and analysis of liver transcriptomes from different genetic types of ducks fed ad libitum or overfed, i.e. Muscovy (*Cairina moschata*) and Pekin (*Anas platyrhynchos*) duck species and Mule and Hinny reciprocal duck hybrids.

It appears that gene expression data in these ducks allow to cluster them in 6 distinct groups, according first to overfeeding and then to genetic type, suggesting that differences in gene expression are more due to feeding effect than genetic type effect. Surprisingly, Mule and Hinny hybrids were clustered together in two different clusters according to feeding (Fig. 2). One could expect great differences in gene expression between these two hybrids as fatty liver production is conducted in Mule ducks and not in Hinny ducks. Our results suggest that not using Hinny ducks is not due to their lower ability to produce fatty liver. In fact the reason is more related to the difficulty to produce Hinny ducks according to a less efficient reproduction in terms of fertility and hatchability when female Muscovy ducks are matted to male common ducks [39]. Our results also show that the overfed Pekin group is widely dispersed on dimension 1 with negative and positive values indicating that Pekin ducks have a lower ability and a greater variability to produce fatty liver. The explanation of this greater variability is not clear but could be the result of selection schemes for fatty liver production, mostly conducted in Muscovy parental lines than in Pekin lines.

Differences in gene expressions between fed ad libitum and overfed ducks were then analyzed. To avoid eventual biases due to heterologous mapping and counting of Muscovy, mule and hinny ducks reads on common duck reference genome, DEG were not determined between all genetic types. They were first analyzed in each genetic type. In such a situation biases if any would be identical between ad libitum and overfed ducks from the same genetic type. Then, these DEG were compared between different genetic types (Fig. 3). It appears that many genes were similarly down- and up-regulated by overfeeding in the four genetic types, indicating that response to overfeeding and hepatic steatosis development involve in some part the same genes in the four genetic types. However, some DEG were also found that distinguish ducks according to their genetic types and responses. When enriched functions associated to DEG were analyzed and drawn as enriched annotation profiles the similarities and differences between the 8 conditions were made more obvious showing common and specific enriched functions according to genetic types (Fig. 5). The clustering of these annotations by semantic similarity made it possible to resume the functions associated to the 611 biological processes GO terms in a more synthetic way, i.e. 9 clusters. As expected, some annotations were related to lipid and carbohydrate metabolism (cluster 2) thus confirming and enlarging previous results obtained in ducks [20–22, 38] and geese [40, 41]. It is interesting to note that together with up-regulation of genes involved in carbohydrate metabolism and lipid synthesis, other genes involved in lipid catabolism were down-regulated, especially in Muscovy, Mule and Hinny ducks (Figs. 7 and 8). These results indicate that hepatic steatosis in ducks involves a lot of genes playing a role in carbohydrate and lipid metabolisms, not only increasing glycolysis and de novo lipogenesis, decreasing lipoprotein assembly and secretion and extrahepatic uptake of plasma lipids as previously documented [16, 19–22], but also decreasing lipid catabolism. It is also interesting to note that together with up-regulation of lipogenesis and down regulation of lipid secretion and extrahepatic uptake, down regulation of lipid catabolism is more obvious in ducks with a higher ability to produce fatty livers, i.e. Muscovy, Mule and Hinny ducks, and less in Pekin ducks, suggesting that inflammation could be lower in these overfed ducks when compared to Pekin overfed ducks. Muscovy, Mule and Hinny ducks thus appear to store more lipids in the liver than Pekin ducks. Conversely, Pekin ducks export more lipids in the blood, uptake and store them in peripheral adipose tissues [42]. Thought different sensibility to insulin could play a role in the differences observed between genetic types with resistance in Pekin ducks, it appeared however that all genetic types were sensitive to insulin [43].

Some other unexpected or less documented functions were also found, including down-regulation of inflammation, stress and immune responses, especially in Pekin ducks (cluster 6.1, Fig. 9), and up-regulation of proliferation (clusters 8 and 9). As shown in Fig. 10, down-regulation of genes involved in immune response and inflammation confirms the results of other studies conducted in waterfowls [41, 44–46]. Knowing the evolution of NAFLD towards more serious liver diseases, we would expect these functions being increased. In the same cluster, down regulation of response to nutrient, exogenous stimuli and insulin were also observed. Resistance to insulin in obese patient is well documented and is linked to type 2-diabetes, hepatic steatosis and other components of metabolic syndrome. We can thus speculate that immune response and inflammation were up-regulated during the first days of overfeeding and were then down-regulated, hepatocytes becoming resistant or insensitive after over-stimulation as observed for insulin response. Analyses of the kinetics of these genes across the entire period of over-feeding would help to address this question. These down-regulations could also be the result of a trade-off between transcription of genes involved in carbohydrates and lipid metabolisms which are dramatically up-regulated and other hepato-specific functions, i.e. expression of complement proteins, which are consequently down-regulated. This hypothesis is reinforced by the fact that albumin, one hepato-specific gene, is also down-regulated after overfeeding ([37] and this study).

Many GO terms associated to proliferation were also up-regulated (additional file, clusters 9.1 and 2). Our results suggest that in addition to hypertrophy hepatic steatosis is also associated to hyperplasia of hepatic cells. Adipocyte hyperplasia is well documented in obesity [47, 48] but as far we know this has not been reported yet in hepatic steatosis. However, further investigations are now needed to confirm hyperplasia in hepatic steatosis. Other studies should also be conducted to complement our results, focusing on non-coding RNAs knowing their roles in the regulation of gene expression.

## Conclusions

Our study is the first report describing whole transcriptomes in four duck genetic types fed ad libitum and overfed. It helps better characterizing responses to overfeeding in ducks in terms of gene expression and associated functions. It also highlights up- and down-regulation of some unexpected functions in duck hepatic steatosis including immunity and cell proliferation. According to duck selection, the results could represent first milestones for early selection in mule’s parents, of ducks with traits of interest to the profession by identifying genes with different levels of expression between genetic types and correlated with their “foie gras” production.

## Additional files

Additional file 1: Experimental and sequencing design. (XLSX 16 kb)

Additional file 2: Gene and transcript annotations. (XLSX 1413 kb)

Additional file 3: Gene counts. (XLSX 8974 kb)

Additional file 4: Liver weights. Ap: Common Pekin duck; Cm: Muscovy duck; mu: Mule duck; hi: Hinny duck. (DOCX 24 kb)

Additional file 5: Clusters of enriched GO terms associated to differentially expressed genes defined by semantic similarity. (PDF 2017 kb)

## Abbreviations

DAVID: Database for annotation, visualization and integrated discovery
DEG: Differentially expressed genes
EAP: Enriched annotation profiles
FA: Fatty acids
GO: Gene ontology
GOBP: Gene ontology biological processes
HC: Hierarchical clustering
NAFLD: Non-alcoholic fatty liver disease
NASH: Non-alcoholic steatohepatitis
NGS: Next generation sequencing
PCA: Principal component analysis
PCR: Polymerase chain reaction
qPCR: Quantitative polymerase chain reaction
RIN: Ribonucleic acid integrity number
RNA: Ribonucleic acid
RNA-seq: Ribonucleic acid sequencing
TMM: Trimmed mean of M-values method
VLDL: Very low density lipoproteins
