# Supplementary material for: RNA-seq analysis of hepatic gene expression of common Pekin, Muscovy, mule and hinny ducks fed ad libitum or overfed
Source: BMC Genomics. 2019 Jan 7;20:13. doi: 10.1186/s12864-018-5415-1 (PMC6323773; doi:10.1186/s12864-018-5415-1)
Supplement: Supplementary file 4 — Liver weights. Ap: Common Pekin duck; Cm: Muscovy duck; mu: Mule duck; hi: Hinny duck. (DOCX 24 kb) [file 12864_2018_5415_MOESM4_ESM.docx]

**Additional file 4: Liver weights**
