# Supplementary material for: RNA-seq analysis of hepatic gene expression of common Pekin, Muscovy, mule and hinny ducks fed ad libitum or overfed
Source: BMC Genomics. 2019 Jan 7;20:13. doi: 10.1186/s12864-018-5415-1 (PMC6323773; doi:10.1186/s12864-018-5415-1)

Cluster 1 (75 GO Terms): Cellular aromatic compound metabolic process

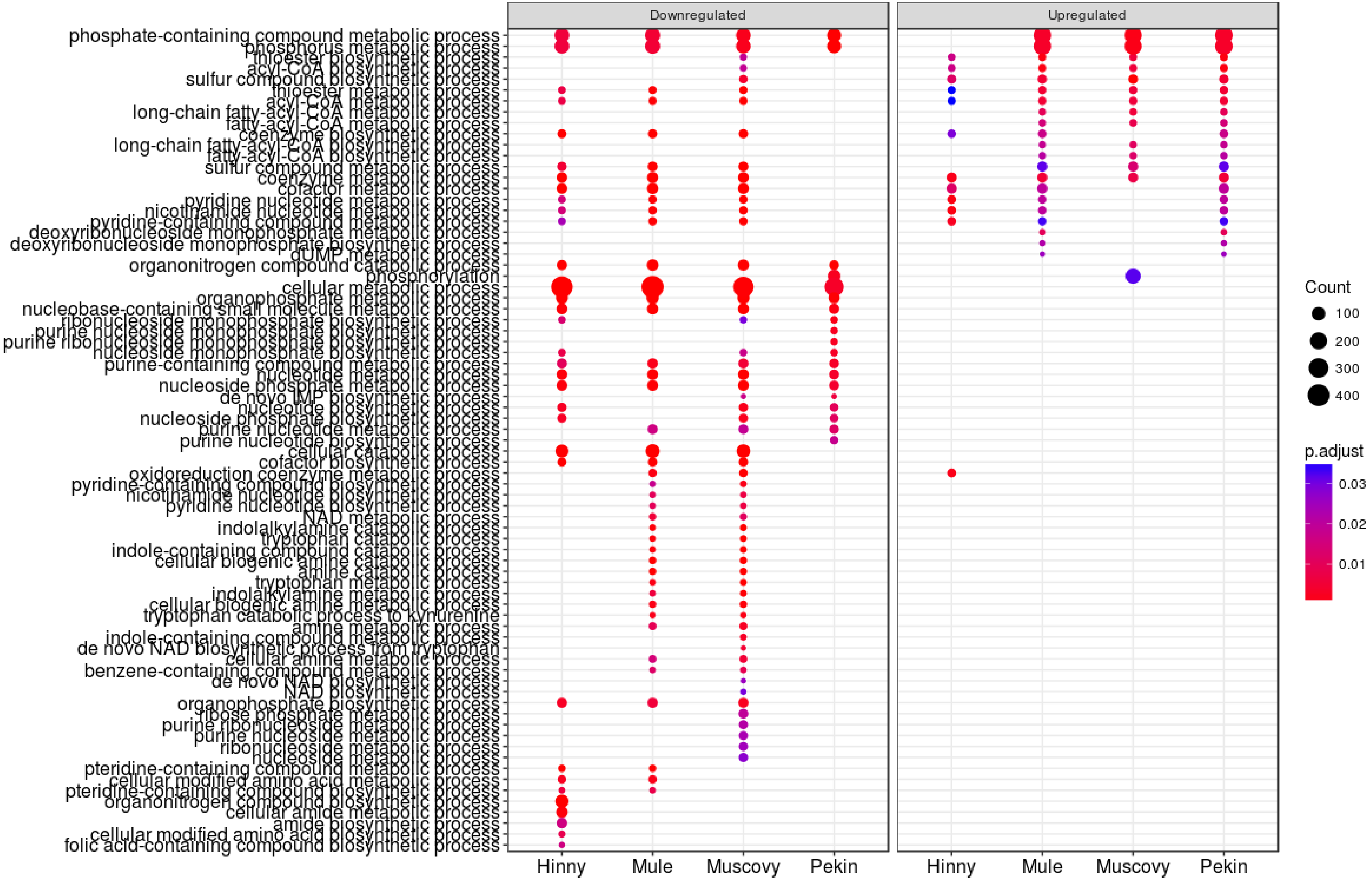

Cluster 2 (108 GO terms): Organic acid metabolic process

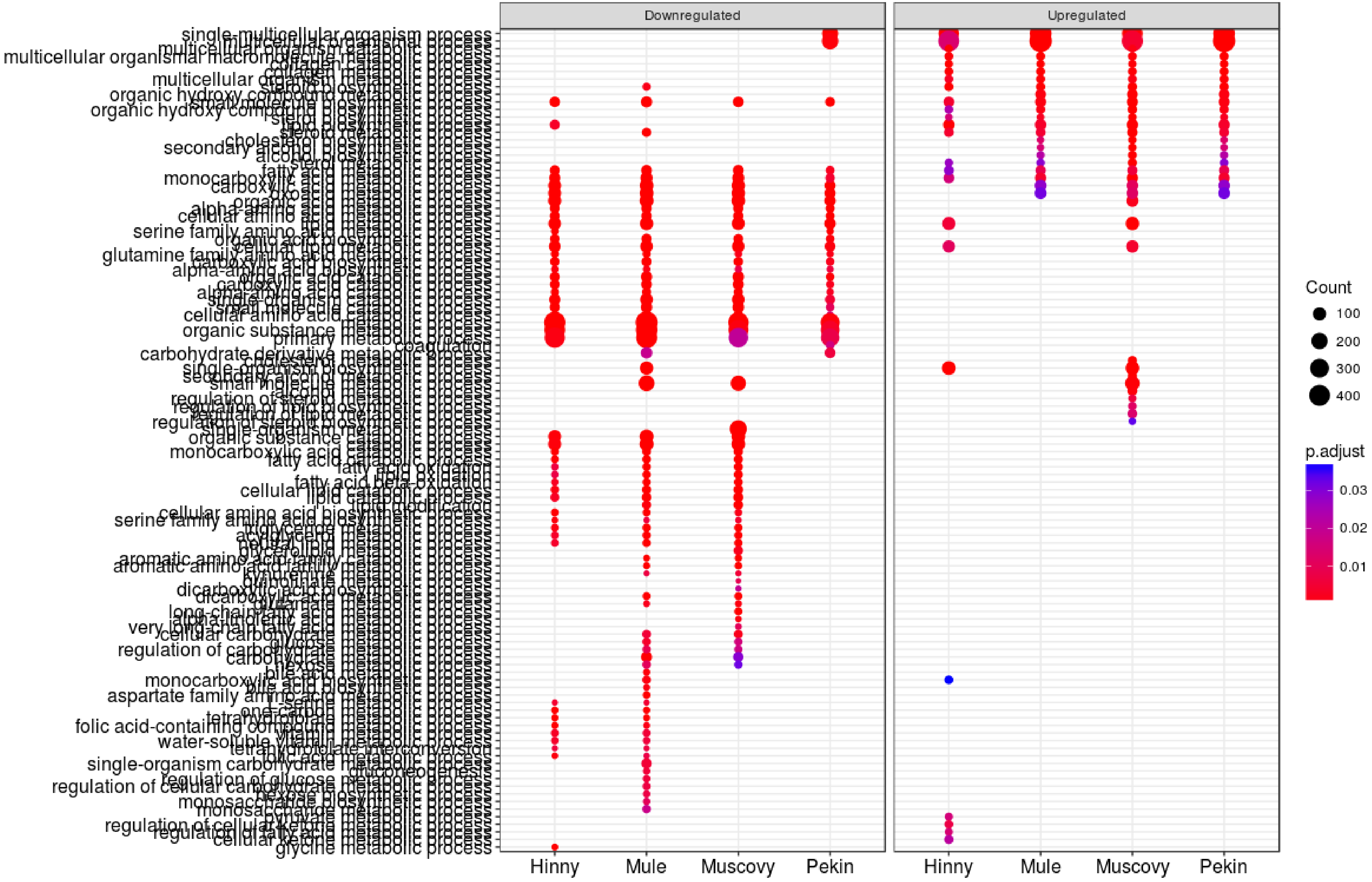

Cluster 3 (83 GO terms), part 1: Anatomical structure development

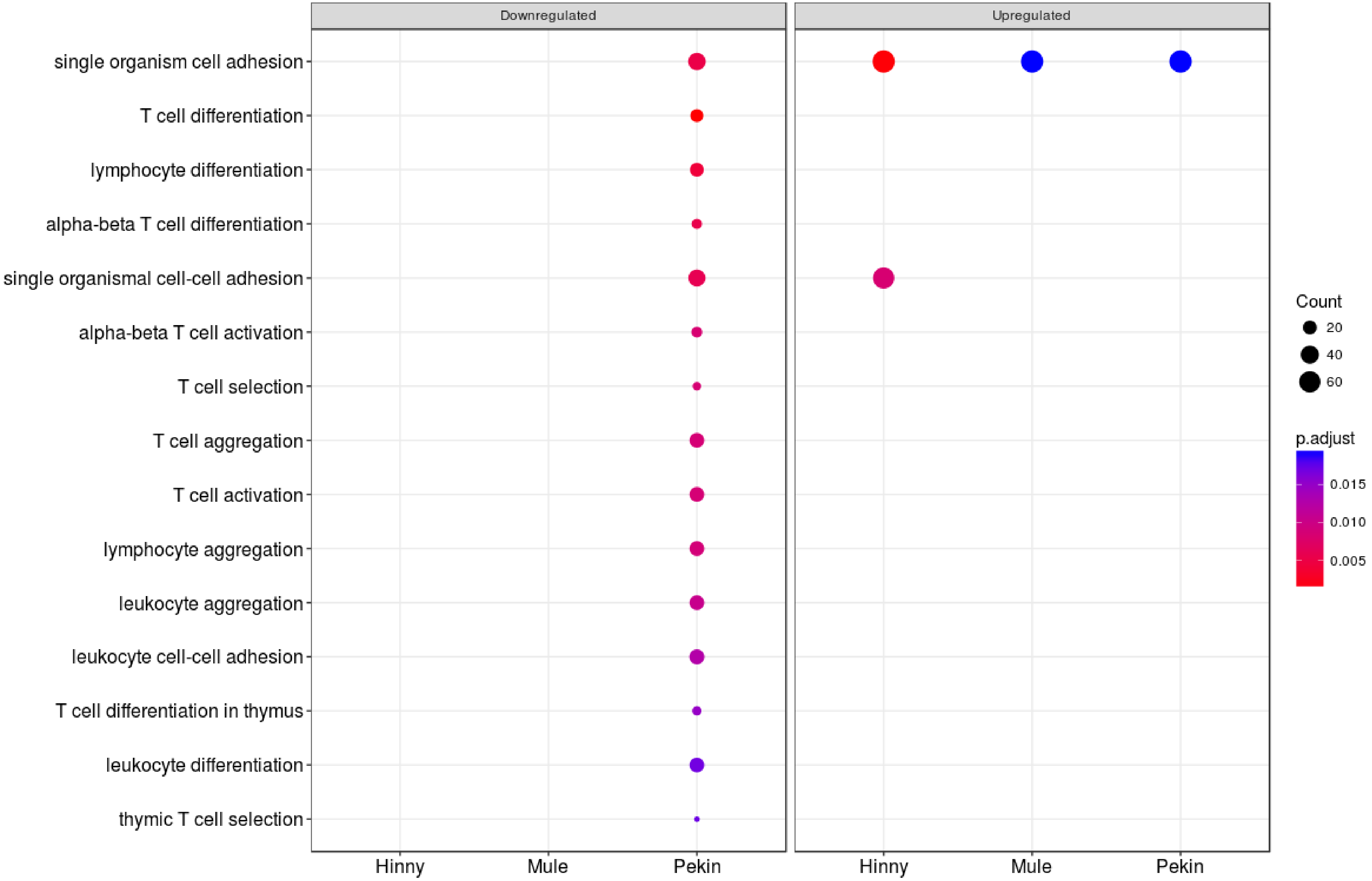

Cluster 3 (83 GO terms), part 2: Anatomical structure development

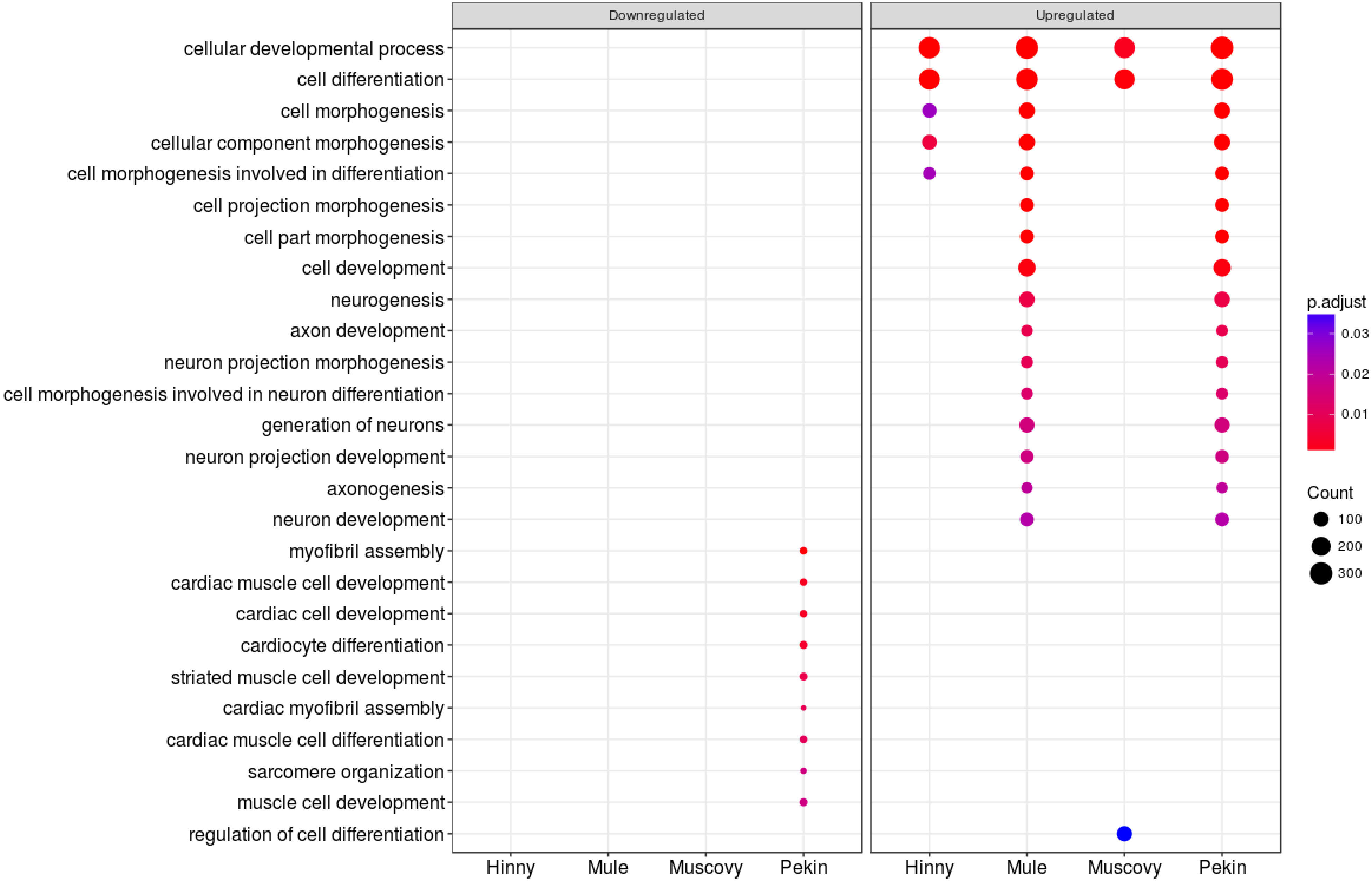

Cluster 3 (83 GO terms), part 3: Anatomical structure development

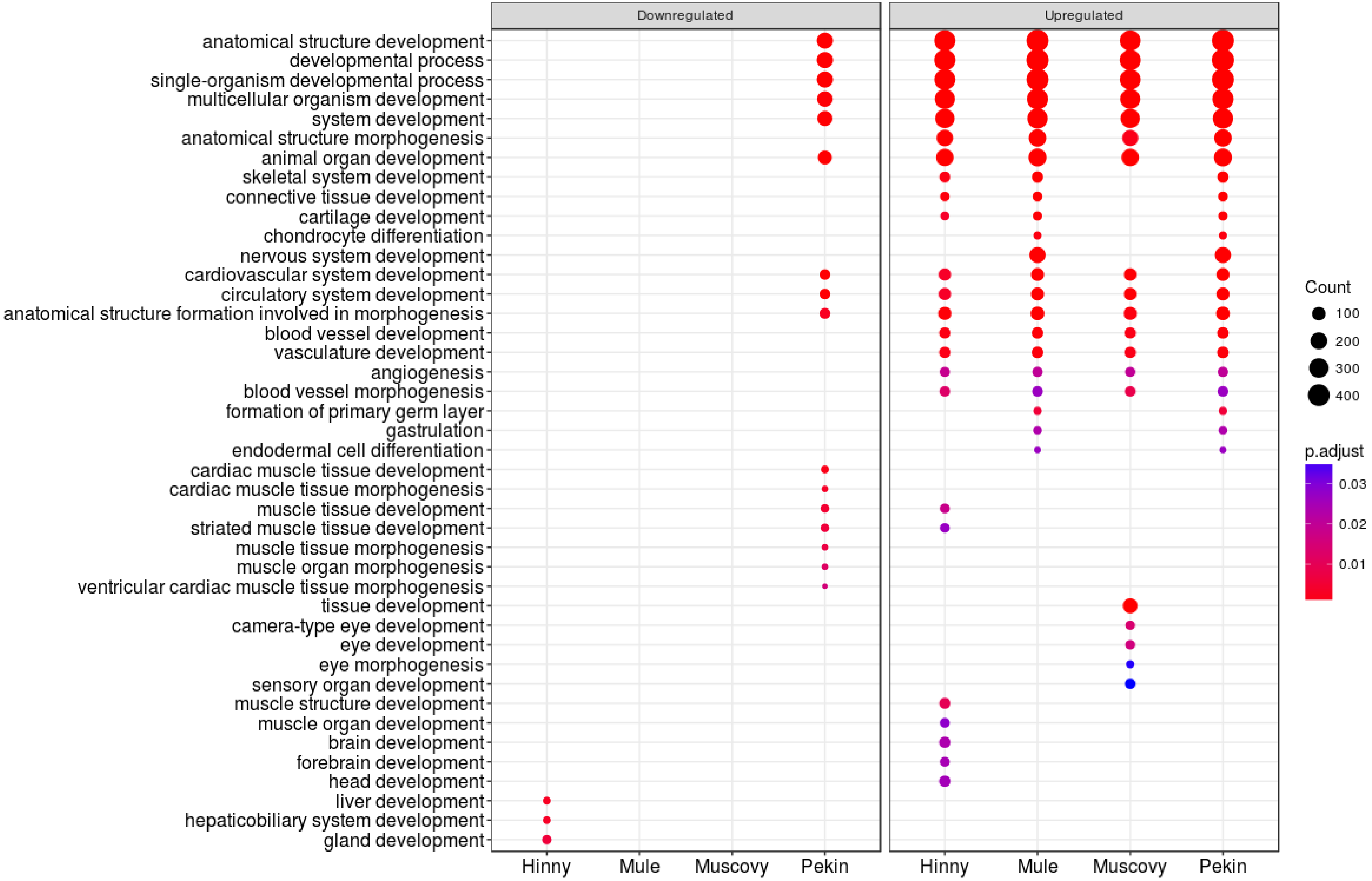

Cluster 4 (42 GO terms): Response to organic substance

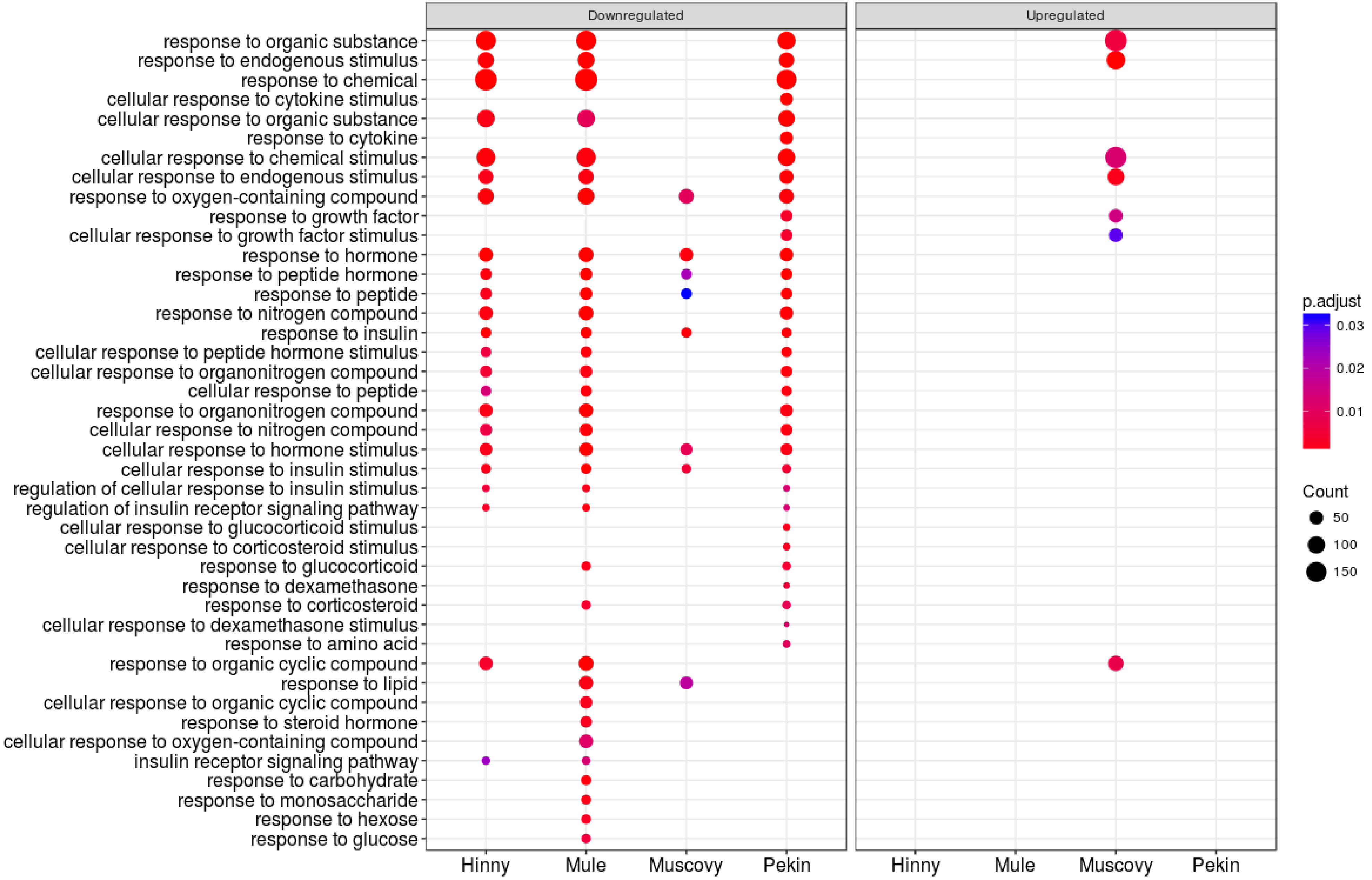

Cluster 5 (40 GO terms): Organic substance metabolic process

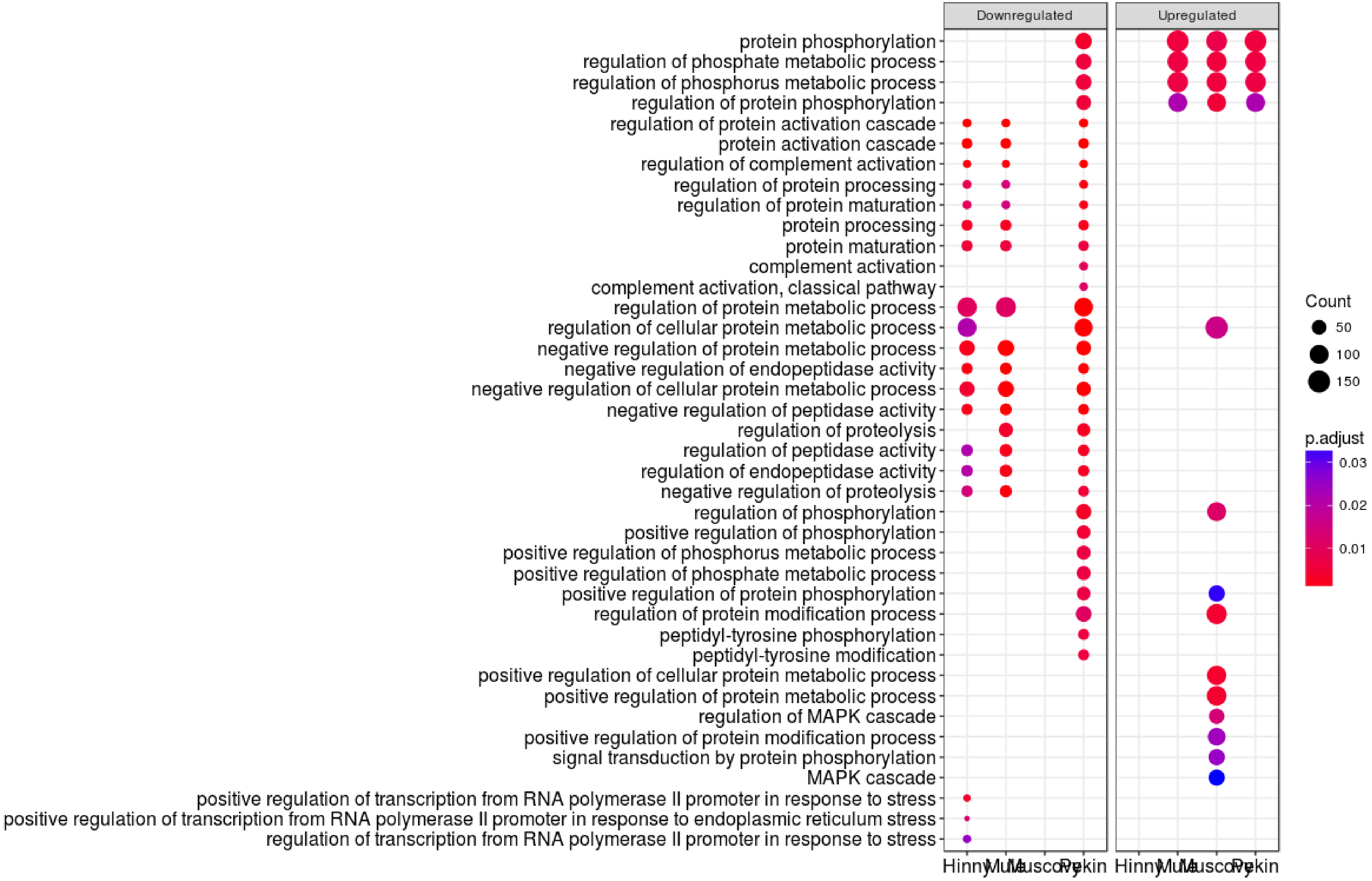

Cluster 6 (99 GO terms), part1: Regulation of biological process

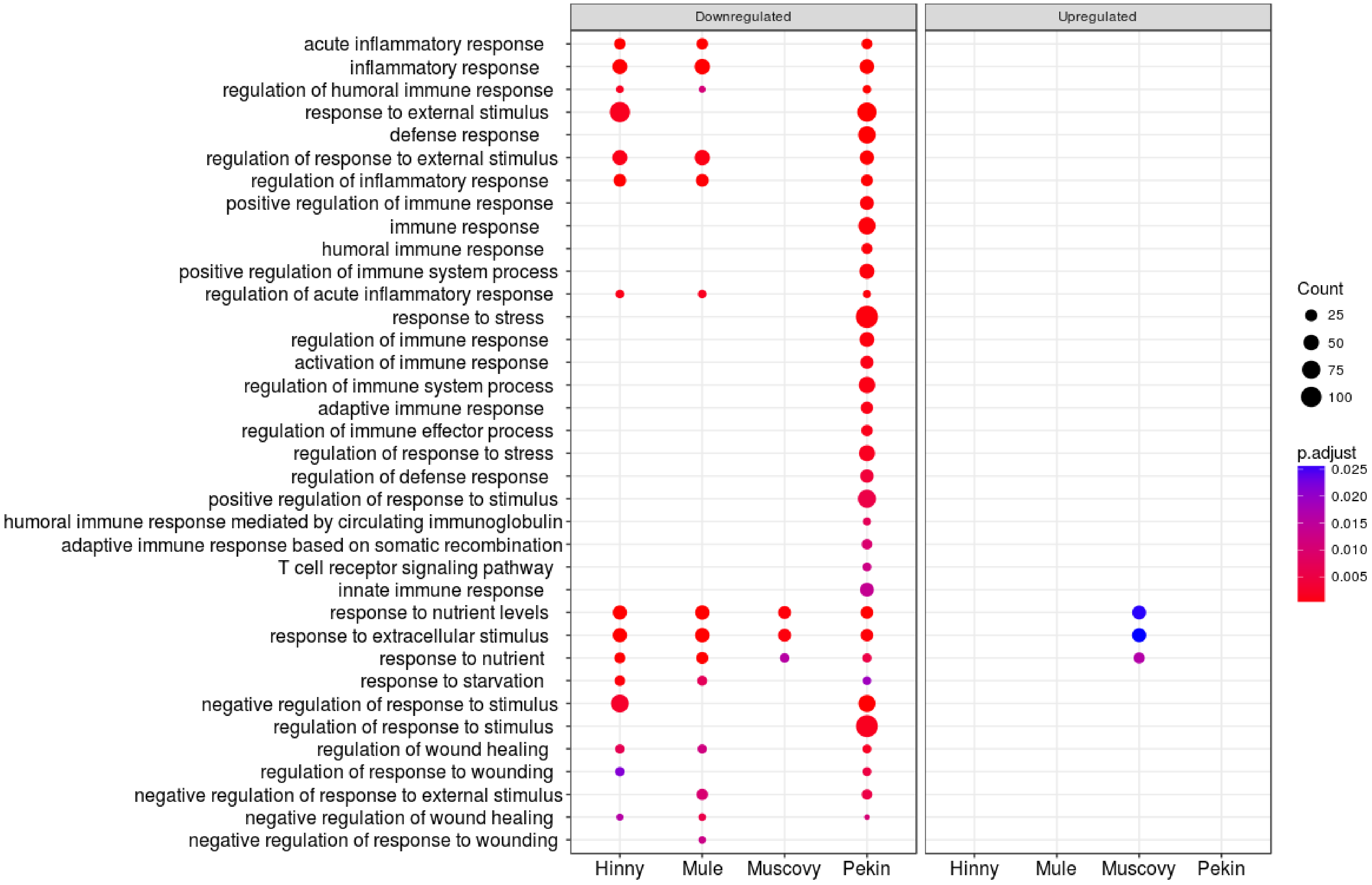

Cluster 6 (99 GO terms), part 2: Regulation of biological process

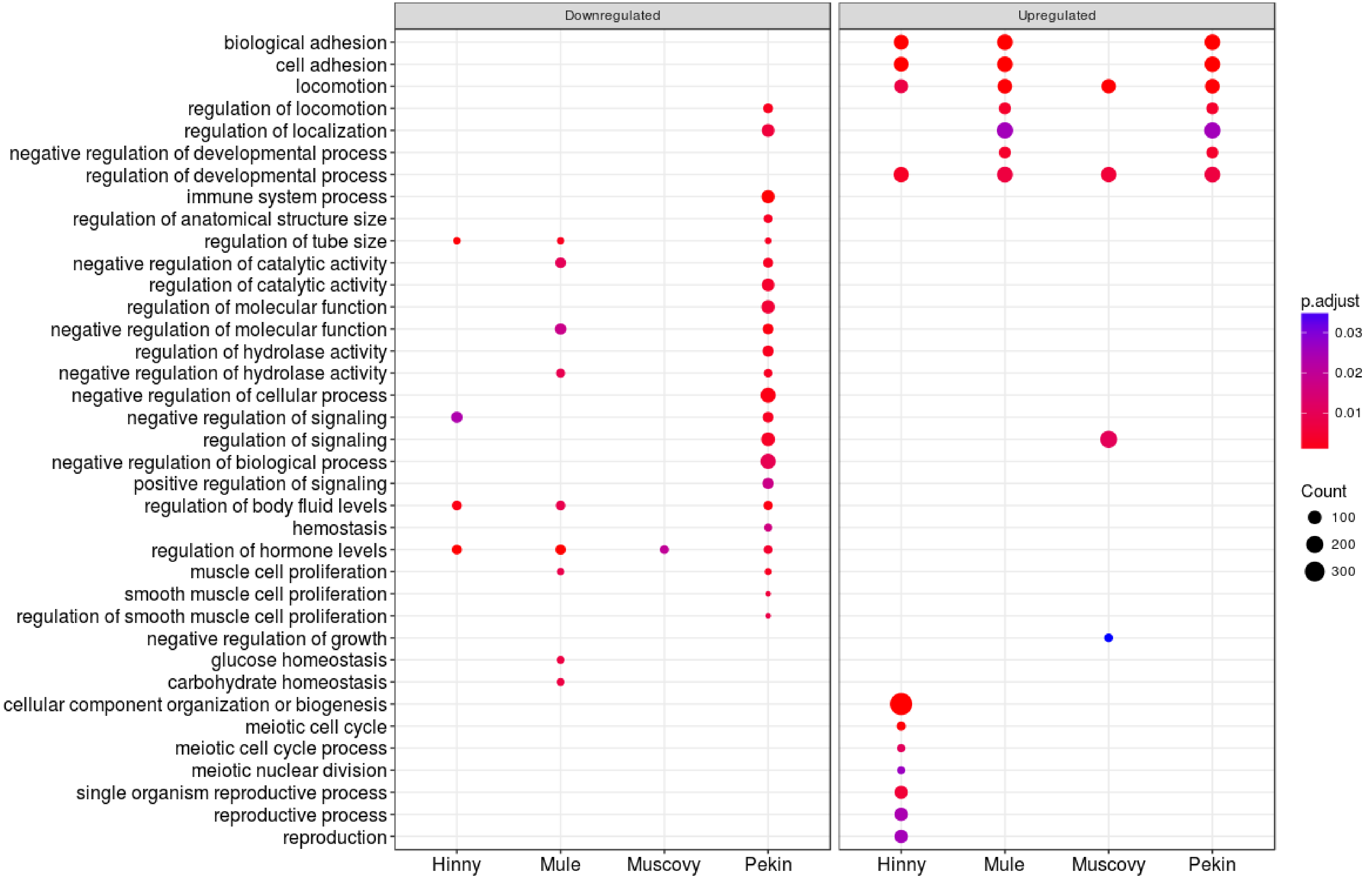

Cluster 6 (99 GO terms), part 3: Regulation of biological process

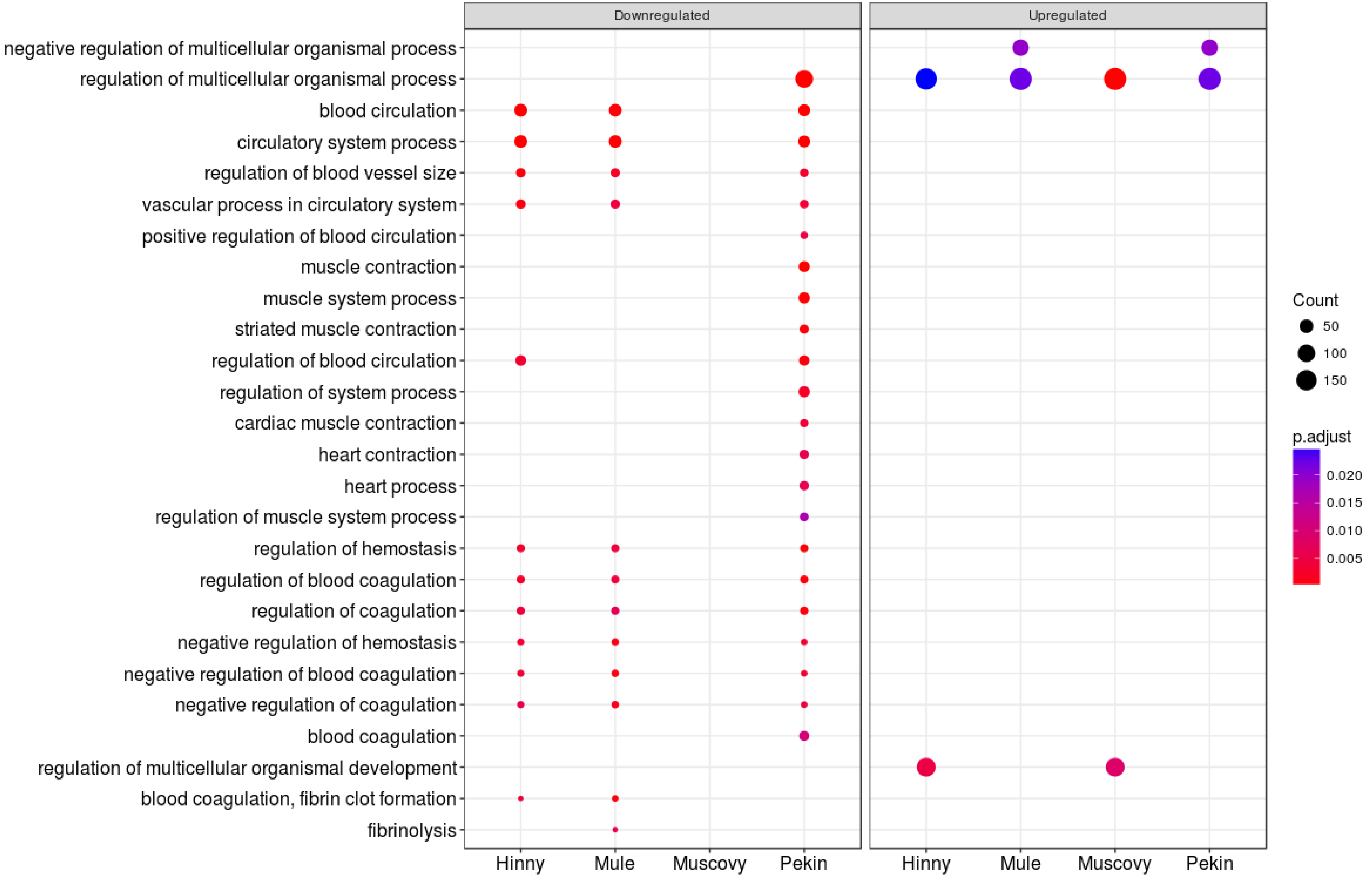

Cluster 7 (41 GO terms): Transport

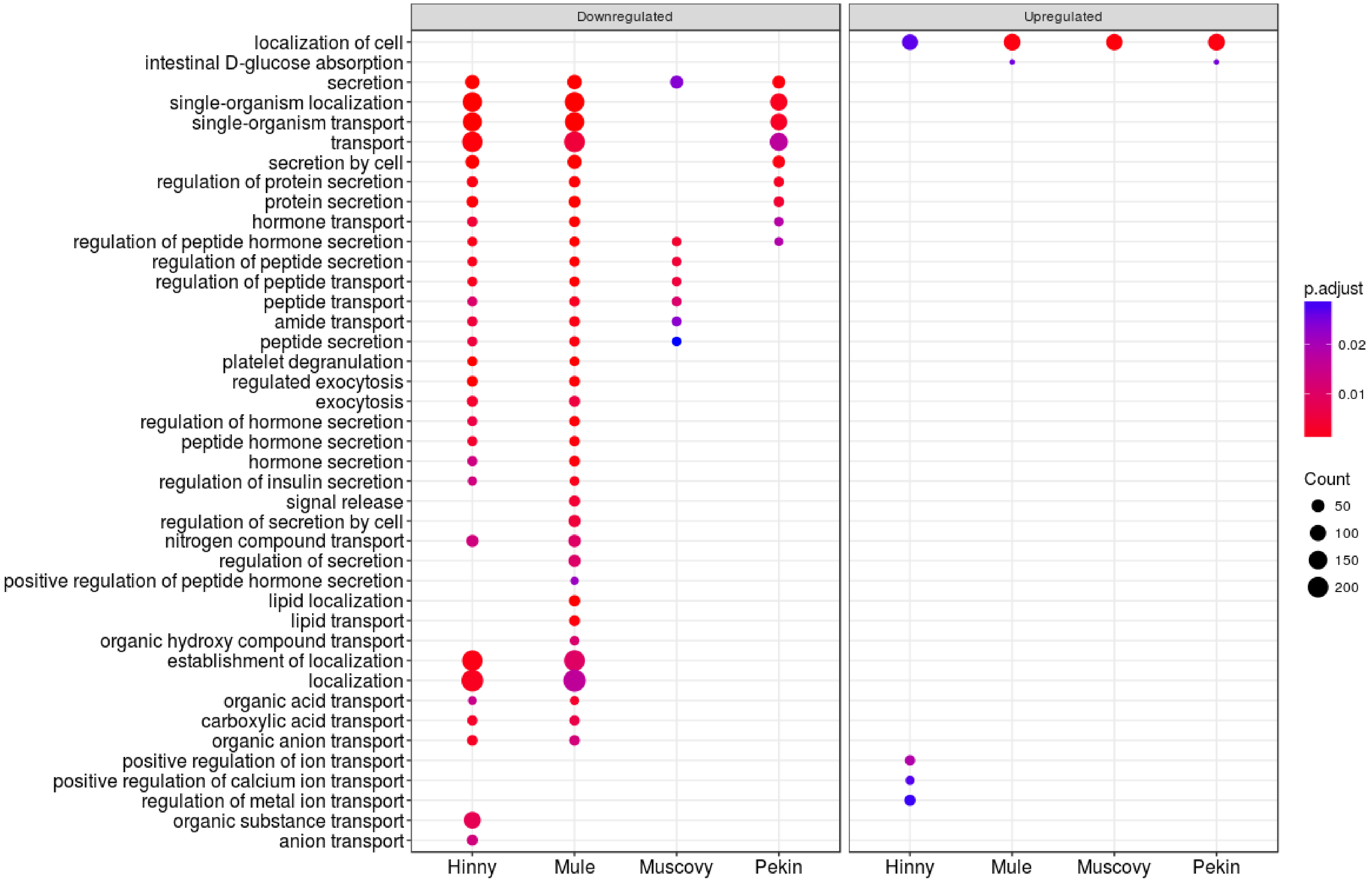

Cluster 8 (36 GO terms): Cellular component organization

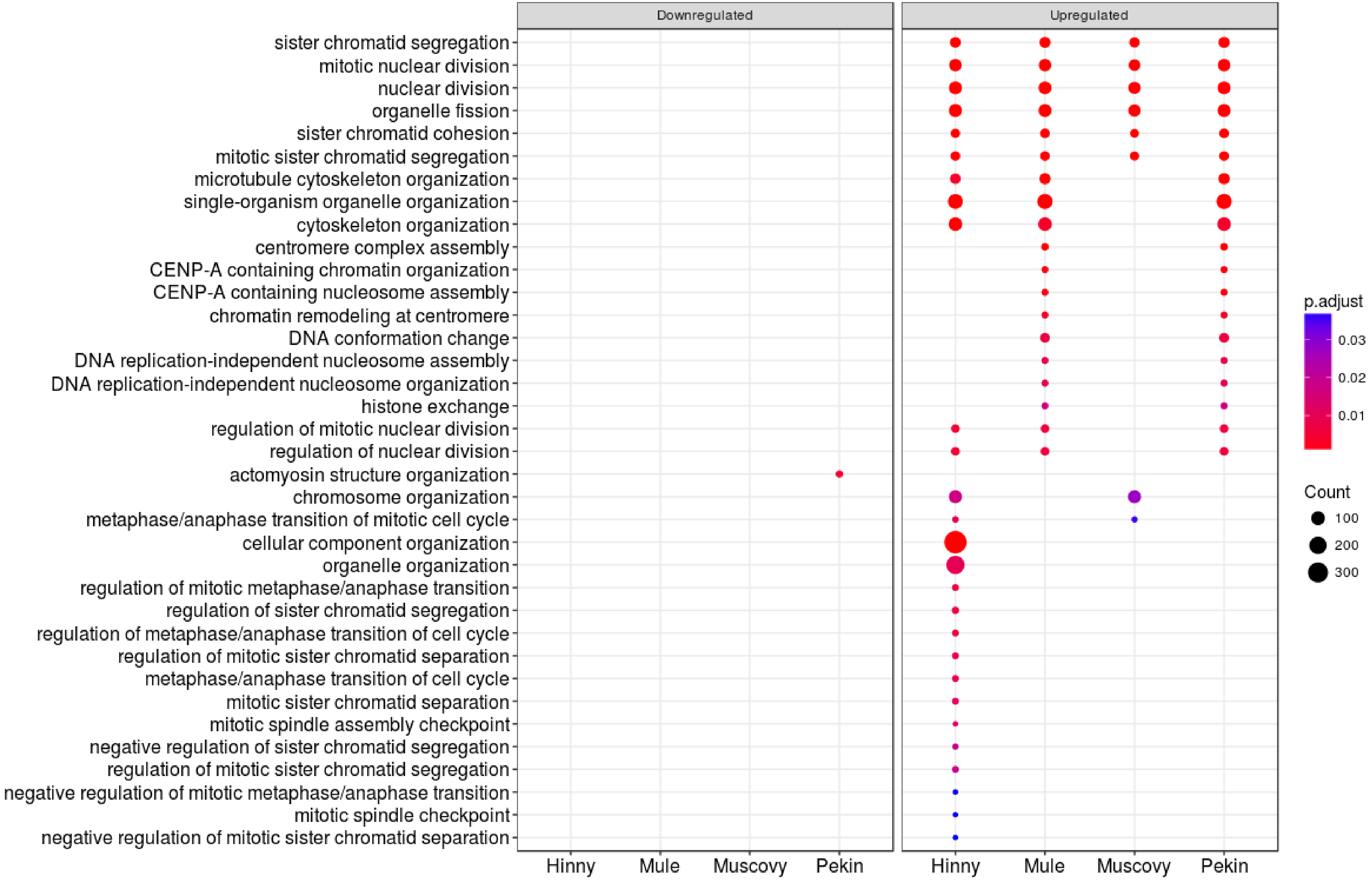

Cluster 9: (87 GO terms) Cell cycle process

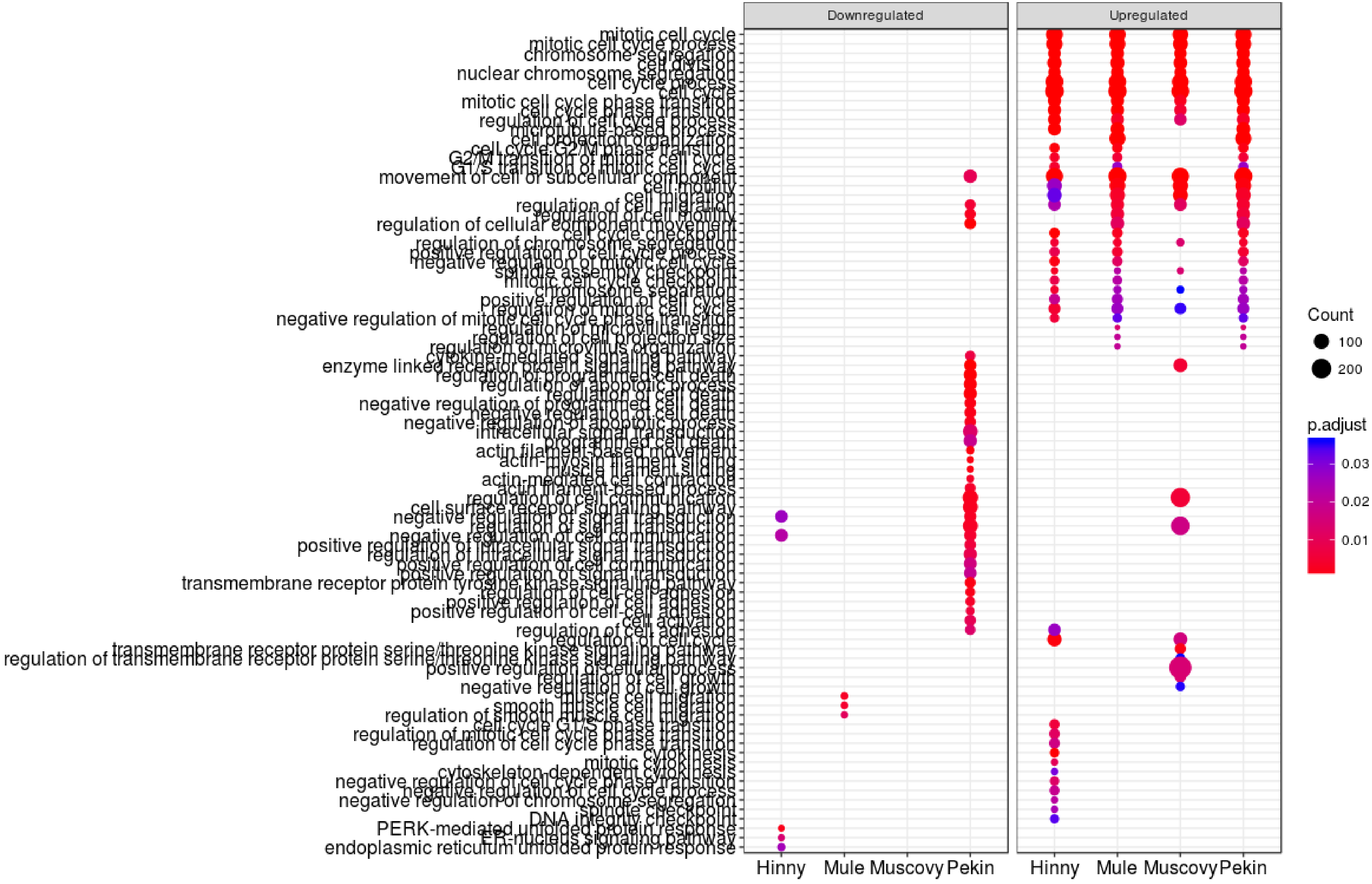

Cluster 9 (87 GO terms), part 1: Cell cycle process

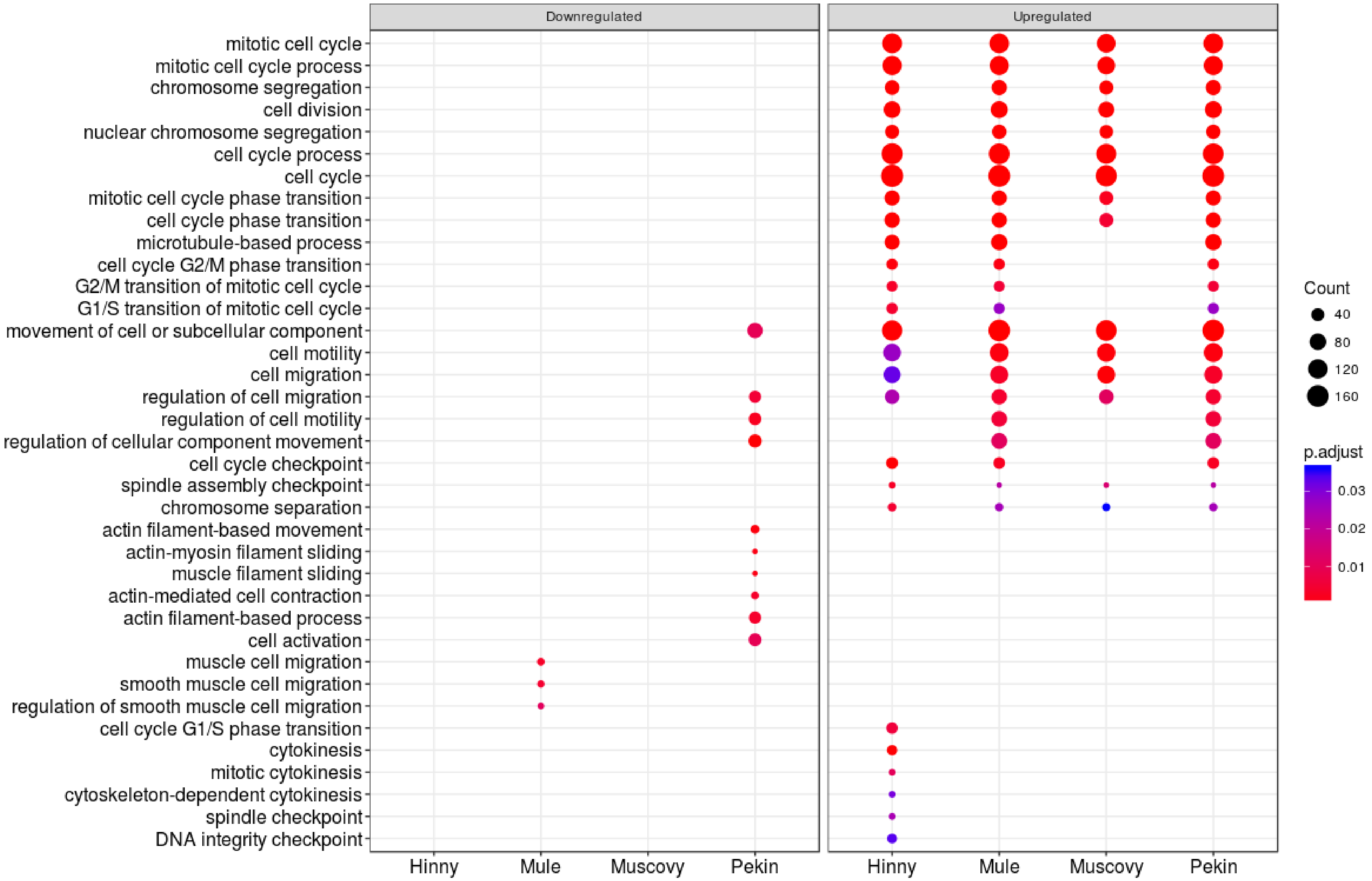

Supplement: Supplementary file 5 — Clusters of enriched GO terms associated to differentially expressed genes defined by semantic similarity. (PDF 2017 kb) [file 12864_2018_5415_MOESM5_ESM.pdf]
